# A Microfluidic Device for Temporally Controlled Gene Expression and Long-Term Fluorescent Imaging in Unperturbed Dividing Yeast Cells

**DOI:** 10.1371/journal.pone.0001468

**Published:** 2008-01-23

**Authors:** Gilles Charvin, Frederick R. Cross, Eric D. Siggia

**Affiliations:** 1 Center For Studies in Physics and Biology, The Rockefeller University, New York, New York, United States of America; 2 The Rockefeller University, New York, New York, United States of America; Duke University Medical Centre, United States of America

## Abstract

**Background:**

Imaging single cells with fluorescent markers over multiple cell cycles is a powerful tool for unraveling the mechanism and dynamics of the cell cycle. Over the past ten years, microfluidic techniques in cell biology have emerged that allow for good control of growth environment. Yet the control and quantification of transient gene expression in unperturbed dividing cells has received less attention.

**Methodology/Principal Findings:**

Here, we describe a microfluidic flow cell to grow *Saccharomyces Cerevisiae* for more than 8 generations (≈12 hrs) starting with single cells, with controlled flow of the growth medium. This setup provides two important features: first, cells are tightly confined and grow in a remarkably planar array. The pedigree can thus be determined and single-cell fluorescence measured with 3 minutes resolution for all cells, as a founder cell grows to a micro-colony of more than 200 cells. Second, we can trigger and calibrate rapid and transient gene expression using reversible administration of inducers that control the *GAL1* or *MET3* promoters. We then show that periodic 10–20 minutes gene induction pulses can drive many cell division cycles with complete coherence across the cell cluster, with either a G1/S trigger (*cln1 cln2 cln3 MET3-CLN2*) or a mitotic trigger (*cdc20 GALL-CDC20*).

**Conclusions/Significance:**

In addition to evident cell cycle applications, this device can be used to directly measure the amount and duration of any fluorescently scorable signal-transduction or gene-induction response over a long time period. The system allows direct correlation of cell history (e.g., hysteresis or epigenetics) or cell cycle position with the measured response.

## Introduction

The utility of time lapse microscopy to study time dependent processes such as the cell cycle and signal transduction has been apparent since the 70's[1]–[3]. The availability of numerous fluorescently tagged proteins[4]–[6], and the ease of integrating digital imaging with software to control the experiments has made this technology more appealing and accessible. Most applications to yeast grow the cells under a gel pad, a quick and easy method which provides fair confinement and a source of nutrients[2]–[3], [7]. However, it has two major limitations : first, continuous tracking of single cells becomes impossible after 5 divisions since cells pile up, so that large-scale and long-term collection of data (for the study of the cell cycle, epigenetic inheritance, adaptation and signal transduction pathways) are compromised. Second, it is impossible to vary the growth media, yet the need to investigate the single cell response to time-dependent stimuli is of major importance in cell cycle research and more generally for yeast cell biology.

Recently, cells attached to a substrate were imaged for long period [8]. This method allows media exchange, but daughter cells may bud upwards and be lost. Yeast have been confined and imaged in microfluidic chambers [9], [10] but rapid control of gene expression was not required for their applications and not demonstrated. Embodying in a single device the capacity for long term imaging and the means to induce and measure transient single-cell responses such as gene expression in unperturbed dividing cells, will greatly improve the resolution of many imaging studies, in the cell cycle context in particular.

Here, we report a novel microfluidic setup for yeast that uses a dialysis membrane to separate the cells from the external flow [11]. It provides much better confinement and planar growth than with gel pads and therefore allows high resolution imaging of single cells for more than 8 division cycles. Dense fields of cells can be segmented from a phase image, leaving the fluorescent channels free. Media can be rapidly changed with no visible movement in the growing colony. We have studied the induction of the regulatable *GAL1* and *MET3* promoters with their respective metabolites, and have shown that externally controlled pulses of gene expression can be achieved with a 10 minutes resolution using the *MET3* promoter. These experiments combined with modeling have allowed a precise quantitative characterization of single-cell responses of these promoters to induction. As a demonstration of the utility of the system for cell cycle studies, we synchronized a growing field of cells to periodic external signals for more than five division cycles, using either a G1/S or a G2/M trigger. These experiments yield quantitative information on cell cycle regulation and variability. The coupling of imaging with a tight control over gene expression will provide accurate and tightly controlled data for any dynamic process with a fluorescent or morphological readout.

## Results

### A microfluidic device to grow single yeast cells as a flat monolayer colony

#### Design of the setup

**Figure 1:**
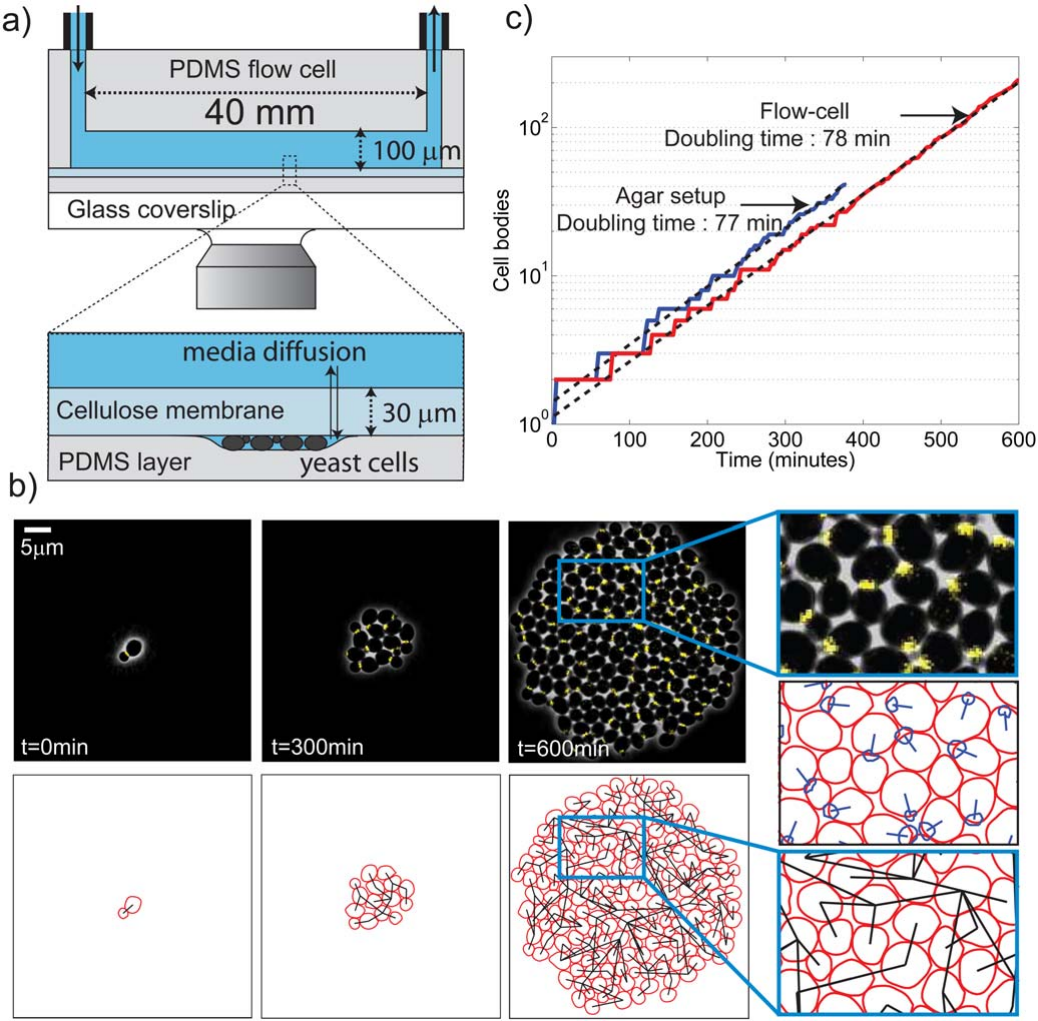
Layout of the microfluidic device; a) sketch of the setup: yeast cells are confined betwen a small layer of PDMS coated on a glass coverslip and a diffusive cellulose membrane (dialysis tubing); the PDMS probably deforms under the cells, thus creating a space between the PDMS layer and the membrane. On top of the membrane, a microfabricated PDMS flow chamber is clamped to control the media; b) Top: Sequence of images showing overlay of phase and YFP fluorescence of a colony of cells that carry the bud neck marker CDC10-YFP during exponential growth; Bottom : cell contours and parentage as retrieved by the annotation software; Right images show enlargments of the cell colony (top), bud neck position (middle), and cell contour and parentage (bottom); c) Growth curves of WT cells during a time-lapse experiment. Data using a standard agar setup (blue line) and the flow chamber (red line) were fit using an exponential (dashed line, discarding the first 100 minutes). The doubling time was respectively 77 and 78 minutes.

We used a diffusive cellulose membrane that separates the growing cells from a main chamber where flow of media occurs[11]: yeast cells were clamped between a polydimethylsiloxane (PDMS) coated coverslip and a membrane, so that they were mechanically constrained to bud horizontally (see Fig. 1a).

#### Chamber stability under flow

On top of the membrane, a 100 micron deep channel (lateral dimensions 40 mm×600 µm) cast in PDMS was connected to media tanks using appropriate plastic tubing. We used a peristaltic pump to drive the flow and electric control valves to switch media (see Fig. S1). We could quickly and reversibly change media without washing away the cells or having to stick them on the surface. A flow rate up to 1 ml/min did not displace the cells nor the imaging focus point, even though this rate corresponds to a high fluid velocity of 0.1 m/s over the membrane and correspondingly large pressures. The setup was mechanically stable over more than 12 hours.

#### Coverslip PDMS coating

Coating the coverslip with a ≈40 µm thick layer of PDMS was necessary to ensure the fast and reliable growth of cells : cells did not grow well when sandwiched directly between glass and the membrane. PDMS is 100 times softer than a yeast cell [12], [13], and therefore cushions the cells, perhaps explaining this effect. E. coli will also grow in our apparatus (data not shown), directly sandwiched between the PDMS and the membrane. In Ref [11] bacteria were confined to groves in the PDMS to faciliate imaging. Evidently this is not required for growth.

Whether nonadherent mammalian cells can be grown in our device has not been investigated.

#### Media switching time

The dead volumes in the tubes were at most tens of microliters, so fluid switching times with our flow rates were a few seconds. Therefore, the effective time to switch media was likely to be limited by diffusion through the cellulose membrane (thickness ≈30 µm and molecular weight cutoff 14 kDa) . The kinetics of fluorescence washout following switch from a fluorescein solution to water was on the order of 30–40 seconds, which gives an estimate of the diffusion time (data not shown).

#### Sample heating

The microfluidic setup was placed on a heated aluminium plate at 32°C (see Fig. S2). The microscope objective was heated in a similar manner in order to ensure that no heat is lost through the objective. In contrast to enclosing the microscope in a heating chamber, local Peltier-based heating-cooling regulation allows efficient temperature regulation over ≈10 degrees within a few minutes.

#### Image quality

Phase contrast and fluorescent images were acquired using a low noise digital camera. The phase images were quite sharp, allowing accurate cell boundary segmentation (see Fig. 1b)[7]. Fluorescent images of subcellular structures tend to be improved over agar confinement, but at the expense of a higher background, due to wavelength-dependent membrane autofluorescence. We used a software-based background subtraction method to remove the membrane contribution to the signal (see Methods).

#### Setup automation

The whole setup was fully automated and driven by Matlab based software: image acquisition; temperature, pump, valves, microscope and stage control, allowing for a fast and versatile control of the time-lapse process. Image processing (background substraction, cell segmentation) could be integrated within the same master program. Therefore, automatic steps in image analysis were done immediately after image acquisition.

#### Growing and tracking single cells over 8 generations

Under these conditions, we were able to monitor proliferation of a single founder yeast cell over more than 8 generations (see Fig. 1b and suppl. Fig. S3 and Movie S1). Cell division time (about 78 min for WT cells at 32°C) was found to be in good agreement with agar pad time-lapse experiments done on our thermally controlled stage (see Fig. 1c) and comparable to liquid culture growth rate (data not shown).

We followed up to 10 separate colonies in an experiment, derived from 10 founder cells found in the flow chamber where about 20 cells were present at the beginning of the experiment. The typical number of cells in the chamber after 12 hours (≈9 divisions) was thus about 10∧6 cell/mL, which is 100-fold below saturation density (the volume of the chamber is few microliters). Therefore, even in the absence of the usual medium flow, no nutrient limitation was expected at the end of the experiment. There are many validated fluorescent markers for phases of the yeast cell cycle. The most useful of these, such as the budneck marker *CDC10*-YFP[7], signal by translocation: Cdc10 localizes to the bud neck at bud emergence and then disperses into the cytoplasm upon cytokinesis. Using this marker we could score budding and cytokinesis even in a dense field of cells, with nearly single frame (3 minute) accuracy. We have further developed our MATLAB-based segmentation and fluorescence analysis software [7] to deal with larger colonies. We could thus monitor the growth and division of cells and construct pedigree trees of microcolonies containing more than 200 cells (see Fig. 1b, Table S1 for timings, suppl. Text S1 and Movie S1).

### Controlled gene expression using inducible promoters

In budding yeast, there is a limited number of effective inducible promoters, among which are the *MET3* promoter (*MET3*pr) and *GAL1*pr. The *MET3*pr is appealing for the temporal control of gene expression since its expression level is not as high as the *GAL1*pr, which generally causes massive overexpression compared to most native promoters; also, the *MET3*pr does not require a change in carbon source (unlike *GAL1)*; the carbon source changes required for *GAL1* regulation can have highly pleiotropic effects on metabolism obscuring specific effects of gene induction. The *MET3* promoter is repressed by methionine (Met) in the media, and there is very little change in growth conditions comparing SCD+0xMet and SCD+10xMet (where 1xMet is 20 mg/L as in standard SCD media[14])[7].

### Kinetics of the *MET3* promoter

**Figure 2:**
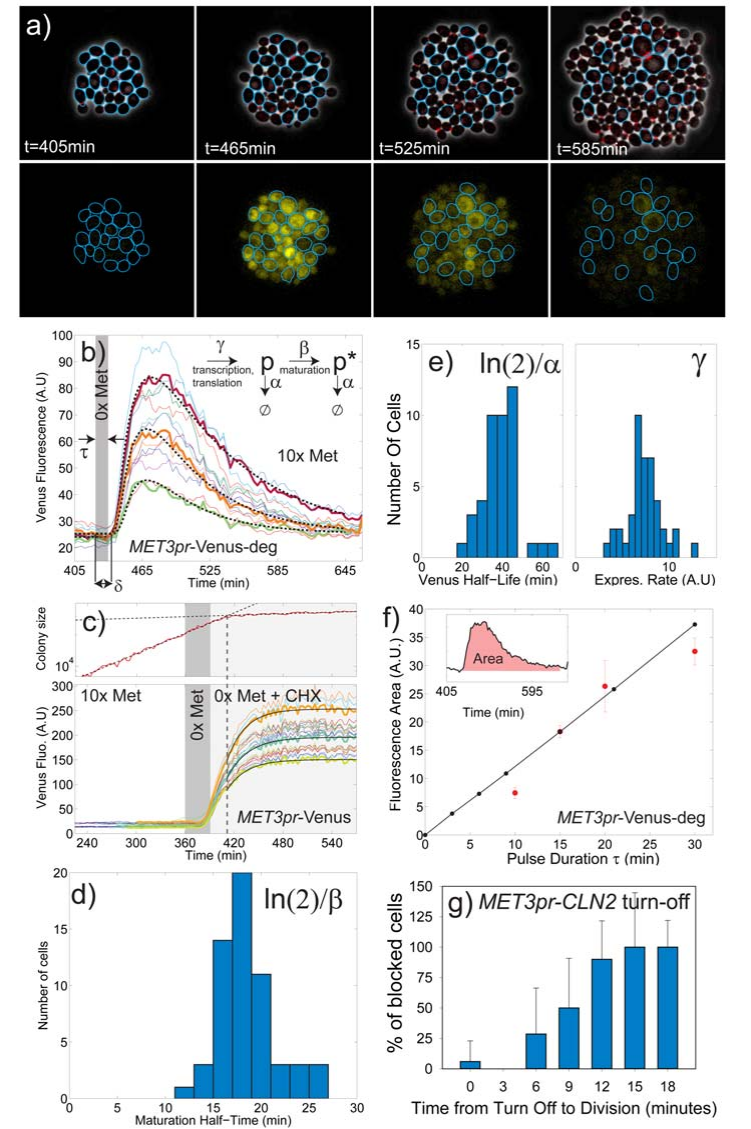
Transient induction of the MET3 promoter; a) Phase contrast, and fluorescence images of MET3pr-Venus-deg cells that were shifted to media lacking Met (0x Met) for 15 minutes at T = 420 min. Blue contours indicate cells whose mean fluorescence is monitored in (b). Top images show the phase contrast overlaid with the MYO1-mCherry budneck marker. Bottom images show the Venus fluorescence in the yellow channel. b) Quantification of mean cellular fluorescence as a function of time after media switch (indicated as grey shade). Each color curve represents a single (mother) cell. Several representative fits of the bold curves to the model (inset, see text) are shown as dashed lines. Inset: protein expression model used to analyze the fluorescence signal (see suppl. model for details). c) Measurement of Venus maturation time in cells carrying stable MET3pr-Venus; Cycloheximide (2 µg/ml) is added to the 0xMet media 30 minutes after MET3pr induction (gray shade). An abrupt cessation of growth ensues 21 min later as defined by the intersection of the two exponential fits (dashed) to the colony growth size (red curve, in square pixel units) in the top panel. Bottom: Colored curves show the kinetics of single cell fluorescence. Solid black lines are representative exponential fits to determine the maturation rate b. d) Histogram of the maturation half-time <Tβ> = ln(2)/β measured by fitting individual cells as in (c) : <Tβ> = ln(2)/β = 18.5+/−0.4 min, COV = 0.16. e) Single cell histograms of Venus-deg protein degradation half-time <Tα> = ln(2)/α and expression rate γ; <Tα> = ln(2)/α = 39.2+/−1.4 min, COV = 0.23, <γ> = 7.3+/−0.3, COV = 0.25. f) Integrated mean fluorescence as function of pulse duration (red dots); black dots are predicted using the model, the mean model parameters, and the imposed pulse duration. g) Fraction of blocked cells in a strain that requires CLN2 expression from the MET3pr after division for cell cycle progression, as a function of the time between the switch to 10xMet media and division. This sets a stringent bound on the turnoff time of MET3pr; see text.

To study induction kinetics in single cells, we integrated *MET3*pr fused to the Venus YFP protein[5] destabilized by the *CLN2* PEST sequence [15], Venus-deg (Methods). We grew a colony of cells in the flow cell in SCD+10xMet, and then switched to 0xMet for a defined period τ to induce expression, and then switched back to 10xMet (Fig. 2 and Movie S2). To quantify both the promoter and the fluorescent reporter, we fit single cell time traces to a minimal model, Fig. 2b and Supplementary Text S1. Induction is very synchronous across all cells with a lag δ (defined from the beginning of the media shift to the onset of fluorescence). The lag could come from Met signal transduction, consumption of cell Met reserves, and the activation of transcription and translation. For each cell we fit a production rate γ for non-fluorescent Venus; a fluorescence maturation rate β (*p* and p* represent respectively the unmatured and fluorescent protein concentration); a protein decay rate α and the lag δ.

These four parameters could not be fit simultaneously from single cell traces since their effects were coupled (especially α and β). To get an independent measurement of Venus maturation time we integrated *MET3*pr expressing stable Venus protein (no degron) and then did a 30 min induction of *MET3*pr followed by the introduction of cycloheximide to block new protein synthesis (Fig. 2c)[8]. Colony growth rate (in area) stopped abruptly 21 min after cycloheximide addition. Actual protein synthesis turn off is known to be faster than 21 min, so it is likely that cells keep swelling for a while after cycloheximide flows in (for example, by vacuolar expansion, see top panel in Fig. 2c). However, the same exponential maturation rate will be found no matter when we begin the fit, provided there is no new protein synthesis and all new fluorescence comes from maturation of previously synthesized YFP. A single exponential fit gave directly the maturation half-time <Tβ> = ln(2)/β = 18.5+/−0.4 min, with low cell-to-cell variability, as expected (standard-deviation/mean or coefficient of variation (COV) = 0.16), Fig. 2d. The illumination level was 3x lower than in Fig. 2b to limit photobleaching; indeed, no fluorescence decay was observed on a 2 hour timescale. We used this mean value of maturation rate in order to retrieve α, γ and δ from the curves in Fig. 2b.

δ displayed very little variability (not shown) and its mean value was 16.8±0.6 min; it did not depend on the pulse duration τ (not shown). Histograms of α and γ for each cell are displayed in Fig. 2e. The mean degradation half time <Tα> = ln(2)/α = 39.2+/−1.4 min (COV = 0.23)) is consistent with previous bulk measurements[15]. It includes the effect of dilution by cell growth and photobleaching (which is negligible). The mean expression rate was <γ> = 7.3±0.3, with moderate cell-to-cell dispersion (COV = 0.25). No substantial correlation was observed between these three parameters, or between each parameter and cell size or the phase of the cell cycle, see suppl. Fig. S4.

Varying the pulse duration τ changed the total amount of protein produced, as measured by the area under the fluorescence response curves, see Fig. 2f. Values found were in good agreement with the model using average parameters extracted for τ = 15 min. Thus, the length of time in 0xMet is a good way to control the total amount of protein induced.

To determine an upper bound for the turn-off time of *MET3pr*, we took advantage of a *cln1,2,3* strain with *CLN2* expressed from *MET3pr*. These cells arrest in G1 unless Cln2 is present after division. Both *CLN2* message and protein are degraded in 5–10 minutes [16] and thus Cln2 is a good readout for the current transcription rate. We grew a colony of cells in 0xMet, shifted to 10xMet and recorded the division time of each cell (as defined by *CDC10*-YFP) relative to the media shift, and their subsequent history. When the shift occured more than 9 (resp. 15) minutes prior to division, 50% (resp. 100%) of the cells arrested (Fig. 2g), implying that they did not have enough Cln2 to enter the next cell cycle. Putting together the various times, implies a firm bound of less than ≈10 minutes for *MET3*pr shutoff following the beginning of the media shift, that is very coherent across the population.

### Graded response of *MET3*pr and comparison to other promoters

To further characterize the potential utility of the regulatable *MET3*pr in various physiological contexts, we needed to determine its response to Met level and its strength relative to other promoters. There have been prior measurements that quantify the steady-state expression of *MET25* and *MET3* promoters in batch culture as a function of Met concentration[17], [18]. However, a concern with these experiments at low Met levels is the consumption of Met by the yeast, and thus gradual induction due to Met depletion (N. Buchler, pers. comm.). In the flow cell, the low density of cells (see above) and continual refreshment of medium means that cells will be unable to significantly reduce available Met even at low concentrations. Moreover, in addition to steady-state levels, the transient regimes following media switch can be precisely characterized by measuring the parameters δ and γ.

**Figure 3:**
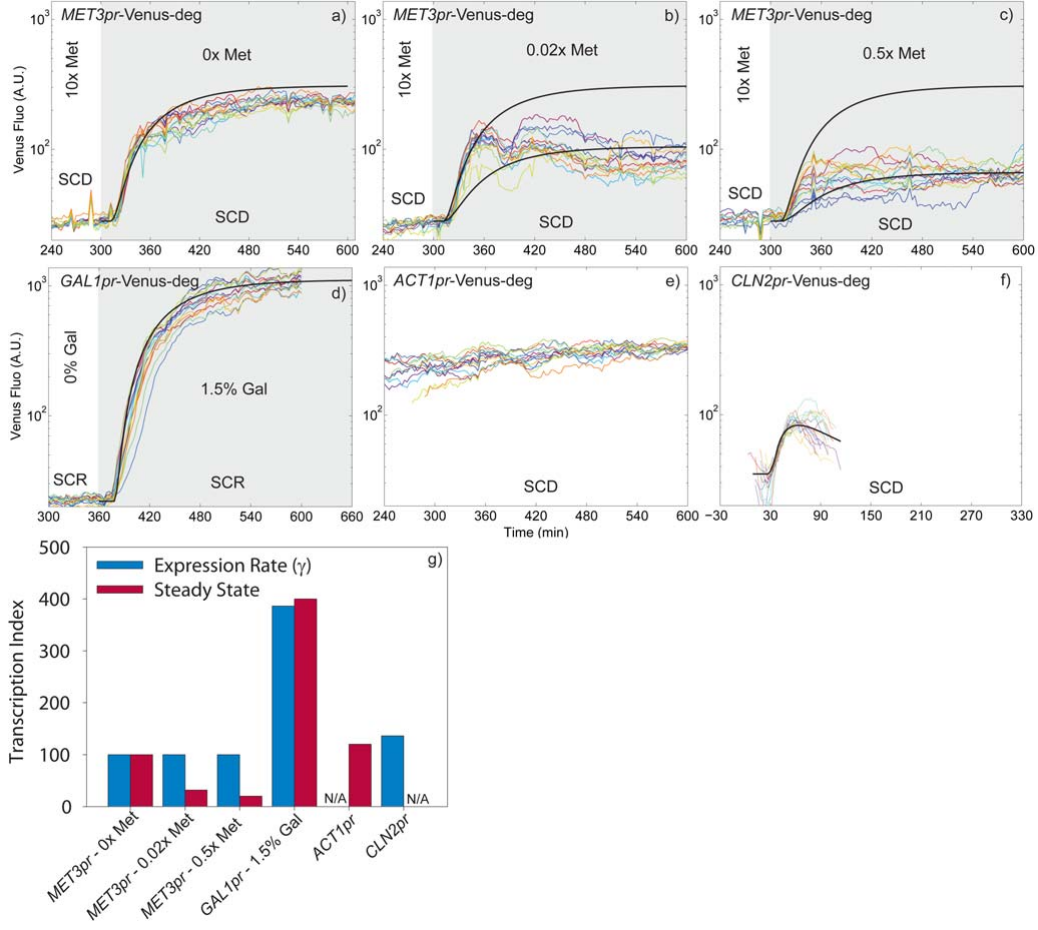
Comparison of expression rates among different promoters; A log scale was used to display signals with different orders of magnitudes; (a-c) Mean fluorescence vs time for various Met induction levels following a media shift. The black curve in (a) is a prediction from the gene expression model fit to the data in Fig. 2, and it is repeated in (b-c) to show that it continues to match the initial increase. The lower black curves in (b-c) are the model with the expression rate γ adjusted to match the asymptotic value (and the other parameters unchanged). d) Induction of the GAL1 promoter following the switch from SCR to SCR+1.5% galactose; The black line is a fit using the same parameters determined in Fig. 2, except γ has been changed to 28; e) Constitutive expression of the ACT1 promoter; f) Expression from the CLN2 promoter. The curves have been shifted to overlay and the black curve is a fit of our model with γ = 10 and an effective pulse duration of 15 minutes. The maturation and degradation parameters are from Fig. 2. f) Comparison of expression rates and steady-state levels for the different promoters, with the MET3 promoter normalized to 100.

We partially induced *MET3*pr by switching from 10xMet to various Met concentrations, see Fig. 3. The model parameters determined for the Met pulse experiments in Fig. 2 describe the shift to 0xMet, with no adjustment. However for shift to low but non-zero Met levels (partial induction), the model describes only the initial fluorescence increase but not the steady state, while if γ is adjusted to reproduce the steady state the initial rise is clearly wrong. Thus the Met regulatory system apparently leads to maximum induction whenever Met becomes limiting to any degree, and then decreases expression, possibly to attain a constant steady-state cellular Met level that is independent of the concentration in the media.

The *MET3*pr strength was then compared to standard promoters : *GAL1*, *ACT1*, and *CLN2*. The same induction experiment as described above was done with *GAL1*pr-Venus-deg cells. The media was shifted from Raffinose (SCR) to SCR+1.5% Galactose.We obtained a good agreement to the data by adjusting the production rate γ to 28 to the steady state level and the time lag δ to 20 minutes (the maturation and degradation rates were unchanged), see Fig. 3d. However, the initial fluorescence rise was not as steep as the model would predict, which may indicate that the *GAL1* promoter activates more gradually than *MET3*pr.

Comparison of the steady-state level of the *MET3*pr at 0xMet to the constitutive *ACT1*pr revealed that the latter was sligthly weaker than *MET3*pr, see Fig 3e. With the highly transient and burst-like *CLN2* promoter, we obtained a good fit to the model by setting γ = 10 and a *CLN2* transcription duration τ = 15 min, see Fig. 3f (γ sets the fluorescence slope while τ defines the peak height). The rate of protein production from the *MET3*pr is thus comparable to the *CLN2*pr, making it a very suitable way to externally control the induction of *CLN2* protein. The comparison among all the promoters is shown in Fig. 3g. Thus, overall, the *MET3*pr displays fast turn on and off times, has rather low cell-cell variability, and has peak expression in a physiological range at least as judged by the comparison to *CLN2*pr. These characteristics make it suitable for physiological experiments in our flow cell.

### Control of cell cycle dynamics using transient pulses of gene expression

The ability to transiently express a gene of interest while simultaneously monitoring cell cycle progression in a controlled environment will facilitate and better quantify many experiments in yeast cell biology. To illustrate the power of our technique for cell cycle applications, we manually triggered the cell cycle by periodically inducing a gene in a strain background where it is required for cell cycling. Since the budding yeast cell cycle is free running and noisy [7], [19], this provided an external clock against which cell cycle transitions can be timed; it immediately generated a large data set for both gene induction and cell cycle response, in a very physiological context. We examined two standard synchronization points, G1/S and mitotic exit.

### The G1/S transition trigger

**Figure 4:**
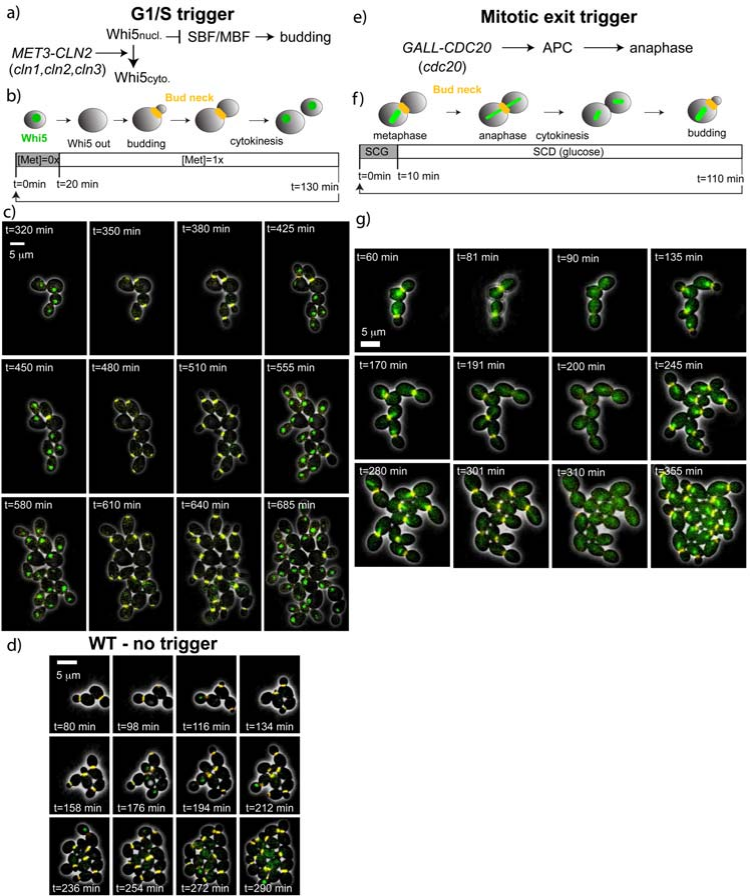
G1/S and mitotic exit trigger; a,e) Sketches of the genetic networks involved in triggering of the G1/S transition and anaphase b,f) The fluorescent markers and design of the periodic pulses c,g) Overlay of phase and fluorescence images during a pulsing experiment. The colors correspond to the schematic in the panel above and the times are chosen to coincide with the events in the schematic. The rows correspond to three successive periods. d) Phase and fluorescence for the growth of WT cells, rows spaced by one doubling-time.

To externally trigger the G1/S transition we used a *cln1,2,3 MET3pr-CLN2* strain which is dependent on *CLN2* expression from the *MET3*pr to activate Start[20]. We used the budneck marker *CDC10*-YFP to time bud emergence and cytokinesis, and added a second fluorescent marker, a *WHI5*-GFP fusion[21]. Whi5 represses the transcription of genes involved in the G1/S transition, Fig. 4a. Whi5 enters the nucleus shortly before division and its exit from the nucleus is the earliest marker available for the Start transition[21], [22]. We measured Whi5 nuclear residence by computing the covariance of the *WHI5*-GFP signal for each cell. The covariance was high when Whi5 was nuclear (i.e. when almost all of the signal was restricted to a small area in the cell), but was low when Whi5 was uniformly spread in the cytoplasm. We used a narrow bandpass GFP filter, which allows better separation of WHI5-GFP and CDC10-YFP.

**Figure 5:**
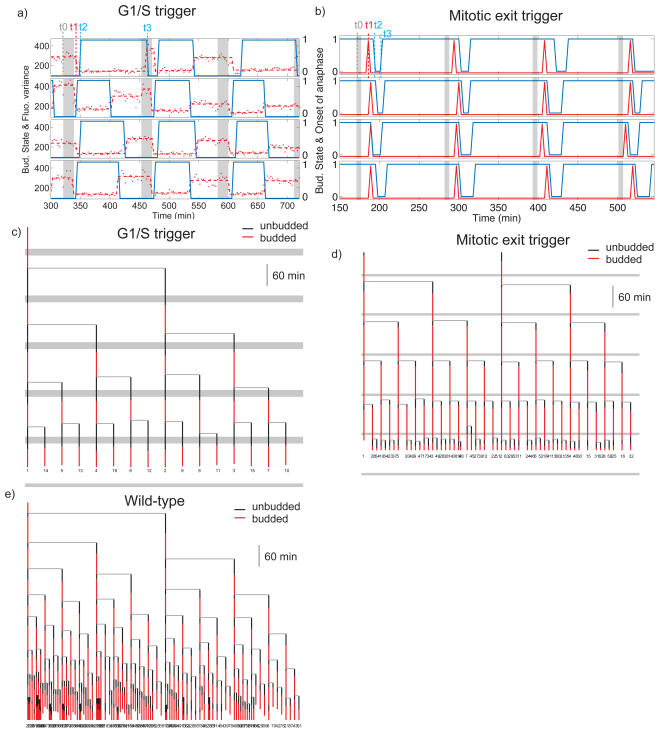
Quantitation of the periodic trigger experiments. a) Time traces of the fluorescent markers used in the G1/S trigger: bud state (blue, 1 is budded) and WHI5 location inferred from the its covariance (red points and dashed line fit), high values are nuclear. The grey band indicates the pulses of SCD+0xMet. b) For the mitotic trigger, bud state is blue, the onset of anaphase is indicated in red, and the SCG pulse is the grey band. c-e) Phylogenies showing the budded/unbudded periods for all descendents of a founder cell, with pulsing bands overlayed where appropriate : c) the G1/S trigger; d) the mitotic exit trigger; e) WT cells.

We transferred cells growing in 0xMet to 1xMet in the device to block them, and then applied sequential pulses of 0xMet of 20 minutes duration, followed by 110 minutes in 1xMet (Fig. 4b). Cells rapidly and coherently responded to the external trigger, by activating the G1/S transition. This sequence of events was quantitatively monitored (see Fig 4c, Movie S3 and time traces in 5a) by defining appropriate times: t0, beginning of induction pulse; t1, Whi5 nuclear exit; t2, bud emergence, t3, division. Strikingly, the cells maintained perfect synchrony for Whi5 nuclear exit and budding during the whole sequence of pulses (3 cycles shown in Fig. 4c); compared to the well-known asynchrony of WT cells (Fig. 4d). The 130 minute clock period is substantially longer than the WT population doubling time of 78 minutes. Cells thus got larger each division since in yeast, cell growth is largely independent of the cell cycle[23]. Synchrony is also evident in the cell cluster phylogeny in Fig. 5c compared with WT cells in Fig. 5e.

The average length of the t0->t1 interval was 18±2.5 minutes in clocked cells. This is in good agreement with our fluorescence based assay for *MET3*pr activation, where the lag δ was similar. Thus the initial ramp up in Cln2 production was sufficient to trigger a sharp Start transition as defined by Whi5 nuclear exit. Therefore, our data place tight bounds on all sources of noise between Met in the media and *MET3*pr activity in a physiologically relevant context.

For comparison to WT cells, we assigned t0 to be the time of the previous WT division, in which case the t0->t1 interval is 11 and 1 minute(s) in WT daughters and mothers respectively. The appreciable WT mother-daughter asymmetry in the t0->t1 interval, was abolished in our clocked cells, since both mother and daughter experienced a synchronous pulse of Cln2 (as opposed to WT cells where G1/S cyclin expression is delayed in daughters). Pooling all mothers and daughters in the clocked population, we found tight control of with a COV of 0.2, whereas the corresponding ditribution in WT daughters yielded a COV of 1.2 (Whi5 exit is too rapid in mothers to accurately measure the COV). Subsequent intervals corresponding to events such as budding (t0->t2) and division (t0->t3) had comparable variability to WT (see Suppl. Fig.S5 nd Table S1), indicating that noise in the cell cycle rapidly caused loss of synchrony, even within a single cycle.

In addition to the 130 minute period cycles described above, we carried out the same procedure with a 78 min period (still with a 20 min 0X Met pulse) that matches the WT doubling time. We found that while mothers remained reliably entrained to the 0X Met pulses, a fraction of the daughter cells (the small ones) had not completed their cycle when the next pulse was triggered : therefore they (and their daughters) failed to bud in response to the next pulse, but they caught up at the next one (data not shown). This finding suggests that small cells, even when forced to bud following a *CLN2* pulse, lengthen the budded period and slow down the progression through the cell division cycle[19].

### The Mitotic exit trigger

*CDC20* is an essential gene that is required to activate the Anaphase Promoting Complex (APC) in metaphase, and therefore controls anaphase and ultimate mitotic exit. *cdc20* mutants thus arrest in metaphase, but can be rescued by *CDC20* under the control of the *GALL* promoter (a truncated weaker version of the *GAL1* promoter) when grown on Galactose (see Fig. 4e). We tested synchronization of a *cdc20* mutant using short periodic induction of *GALL-CDC20*, by monitoring the bud neck marker *CDC10*-YFP and a *TUB1*-GFP (beta-tubulin) fusion in order to monitor the state of the spindle during mitosis.

We briefly grew the cells in SCD so they all arrested in metaphase, which was characterized by a short, bright microtubule spindle close to the bud, Fig. 4g. These arrested cells were then placed in the flow cell, and periodically pulsed for 10 minutes in SCG followed by 100 min in SCD, for a total period of 110 minutes, see Fig. 4f. For more than 5 cycles (of which 3 are shown in Fig. 4g, see also Movie S4), the cells divided rapidly after the galactose pulses.

We scored the onset of anaphase occuring at by visual inspection (monitoring the elongation of the spindle) and measured division and budding timings (resp. t2 and t3) using the budneck marker. Fig. 5b displays typical recordings showing these sequences of events (see Fig. S5 and Table S1 for histograms and timings). The cells reached anaphase 15 minutes after the beginning of the pulse with good synchrony (COV = 0.2). A cluster phylogeny in Fig. 5d further illustrates the very good synchrony of the whole micro-colony. In addition, we observed that an SCG pulse as short as 2.5 minutes allows 80% of the cells to enter anaphase, with similar timing (data not shown), showing very sharp induction of anaphase. In contrast, the unbudded period immediately following this synchronous mitosis exhibited significant variability. Thus, these experiments confirm noise in the unbudded period[19]. Comparison to the G1/S trigger experiment shows that this noise can be suppressed by tight induction of *CLN2*, but not by making the preceeding mitosis very synchronous.

## Discussion

Our flow cell design offers several advantages over other designs for long-term imaging of the cell cycle. It is simple to build and uses PDMS technology only to fabricate the flow chamber. Size tolerances are not critical and all the parts can be reused, since we assemble our cell with a mechanical clamp rather than sealing the PDMS to the glass. An unexpected benefit of the membrane confinement is a clearer phase image, and better imaging of subcellular fluorescent markers than what was possible with the agar setup. The two inpenetratable yet elastically deformable layers confining the yeast force almost all cells to bud in the plane of focus. Remarkably, these constraints have no effect on the doubling time compared to spatially unconstrained growth in liquid medium.

The fast and reliable media switching offered by microfluidic devices allows for precise control of gene expression in single cells; this advantage has not been exploited previously. This application is most favorable with promoters than turn on and off quickly. The *MET3* promoter is shown here to exhibit excellent characteristics for such experiments. Induction timing is very homogeneous across the population, although the expression rate varies somewhat from cell to cell. Partial induction is possible with no appreciable increase in cell-cell variability. The promoter turns off in less than 10 minutes. We have also shown usability of galactose in the flow cell to induce the commonly used *GAL1* promoter; in other experiments we have also demonstrated efficient induction with steroid hormone receptors (data not shown)[24]. Thus it is likely that most small-molecule inducers will work in this system.

Control of both the expression level and the timing is essential if an inducible gene is to substitute for a natural gene. The *MET3*pr expresses in a reasonable activity range. It is comparable to *ACT1*pr and 1/4 the level of the other widely used *GAL1*pr, which is sometimes too strong. The output from the naturally very dynamic *CLN2* gene can be recapitulated by maximal induction of *MET3*pr for 15 minutes, with a cell-cell variability of about 25%. Other evidence that *MET3*pr operates in a physiological range comes from the observation that when fused to *CLN2* in a *cln1,2,3* strain, all the cells arrest when grown in 1xMet, but they all progress when induced for 20 minutes in 0xMet.

The efficacy of our device for controlling the cell cycle is manifest in the periodic block-release experiments using either a G1/S or mitotic arrest. We could then time downstream cell cycle events relative to the external clock and thereby better isolate the sources of variability. The flow cell obliviates the need for synchronizing bulk cultures whenever a fluorescent readout is available, and does so with virtually no perturbation in the bulk growth rate.

There is a natural follow-up to the observation of nearly perfect induction of cell cycle synchrony using brief pulses of *CLN2* or *CDC20* in *cln1,2,3* or *cdc20* backgrounds. One may ask if such pulses can also entrain the cell cycle in a wild-type background, and under what conditions. This “mode-locking” experiment, analogous to jet-lag or the phase-response curve in circadian studies, has been examined theoretically using a computational model for the cell cycle [25], [26]; our device now allows an experimental realization.

Other cell cycle questions that we are addressing include the question of whether if we put a cyclin such as Cln2 under the control of the *MET3* promoter, do its targets respond to the integrated gene dosage or its maximum? Is there a hierarchy of targets, that are triggered as the Cln2-dependent kinase level increases? How sigmoidal is the response? How tight is the temporal correlation between the early transcriptional events in Start and sensitivity to mating pheromone? Do the events downstream of Start (e.g. bud emergence, spindle pole body duplication, DNA replication) display different sensitivities to cyclin dosage? If we supply more mitotic cyclin in G2 will mitosis run faster?

Comprehensive genetic screens and genome wide expression data sets provide hints of the complex networks that underlie cellular function. Flow cell technology and time lapse imaging of single cells are essential for performing well-controlled dynamic measurements. We have demonstrated a practical way to follow multiple fluorescent markers, and construct complete cell pedigrees over many cycles of division and growth comparable to conditions in liquid culture, combined with the ability to pulse with inducers. Any pathway that can be activated by small molecules with a response that can be monitored by fluorescence will benefit from our approach. The ability to measure responses in cells for which the complete history and cell cycle positions are known allows detection of epigenetic effects (e.g., hysteresis, ‘memory’ of previous responses) and cell cycle regulation of the response, without the need to purify or synchronize populations, and all manipulations are carried out on otherwise unperturbed, exponentially growing cells.

## Methods

### Strains and plasmids

#### Strain constructions

All strains were constructed either by transformation (see below) or by mating and tetrad analysis, using our standard lab stocks (all W303 background) as starting material (See Table S2 for the list of strain)

#### Plasmids carrying transcriptional reporters

All the plasmids carrying the coding sequence of Venus under the control of a given promoter (*ACT1*pr, *GAL1*pr, *CLN2*pr, *MET3*pr) were made in the following way: the plasmid pJB04T [7] (made from pSVA17[15] containing the GFP sequence fused to the PEST sequence of the *CLN2* (deg) gene and driven by the *ACT1* promoter was cut using PacI and BsrGI and the GFP insert was discarded. We swapped in the Venus insert cut from pKT90 (EUROSCARF, set 1 of optimized tagging cassettes). The resulting plasmid (containing *ACT1*pr-Venus-deg was named pGC04D. The plasmid containing the *CLN2* promoter was made by swapping the promoter of pSVA17 and the one of pGC04D (using BamHI and PacI sites), resulting in pGC08D. Plasmids carrying the *GAL1* or the *MET3* promoter were built as follows : the promoter sequences were amplified using PCR from a template (respectively p405*GAL1* [17] and pRS426-*MET3*-HA-Hog1 (a gift from the Engelberg lab, University of Jerusalem, Israel) for *GAL1* and *MET3*.

#### *MYO1*-mCherry fusion protein

We used the mCherry tagging plasmid (pKT355) donated by the Bi lab (University of Pennsylvania, USA). We designed specific oligos to amplify the coding sequence with 40 nucleotides flanking regions (see primer sequence in Table S2) in order to proceed with a single step PCR gene replacement. Insertion was screened by PCR and fluorescence visual inspection.

### Growth conditions and media

Before starting the time-lapse experiments, cells were pregrown overnight in standard SCD with the appropriate Met dosage. The standard 1xMet concentration was set to 0.02 g/L[14]. The temperature was set to 32°C in the microfluidic setup, which ensured a growth rate very close to the optimal one defined in liquid cultures.

### Microfluidic device

The microfluidic device, as reported on Fig. 1, is made by assembling three components: 1) A PDMS coated coverslip; 2) A cellulose membrane; 3) a PDMS flow cell. The components were prepared as follows:

#### PDMS Coated Coverslip

The PDMS-coated coverslip was prepared as follows: 30 g of PDMS (Sylgard 184, GE) was mixed to the appropriate curing agent ( ratio 1∶10 ), and degassed on the bench for 30 minutes. Then, 50–80 microliters were deposited on the surface of a clean 4 inch silicon wafer pre-coated with trimethylchlorosilane (Sigma Aldrich). A cleaned glass coverlip (gauge 1.5, whose thickness is about 170 microns) was put on top of the PDMS droplet; we allowed the PDMS to spread evenly on the surface of the coverslip for about 30 minutes. The wafer with the coverslip was then baked for 45 minutes at 80°C. The coverslip was then peeled off slowly using a razor blade or scalpel. The typical thickness of the PDMS layer is around 30–40 microns ( coverslip size : 24 mm×50 mm). This extra layer must be smaller than the objective free working distance (which is 100 microns in our case). It did not affect the quality of the imaging.

A coated coverslip can be used for many experiments. We washed them with detergent (Triton, 20 %) and deionized water. The surface of the PDMS tends to become more and more hydrophilic, so that the natural tendency of cells to stick on the surface decreases with time (after ≈10 experiments).

#### Cellulose membrane preparation

The cellulose membrane used is taken from dialysis tubing (Sigma Alldrich, D9527). The high cutoff size (14 kD) allows media molecules to diffuse freely through the membrane pores. The tubing was cut to an appropriate size (22 mm×50 mm) , then soaked in deionized water in order to separate the two sides of the tube of membrane. The cellulose membrane was first boiled in a 2 % w./v. Na2CO3 solution for half an hour to remove glycerin and sulfur compounds, and boiled a second time in TE at pH8 for half an hour. Membranes can be stored wet at 4°C, and reused after cleaning.

The thickness of the membrane (about 30 microns) ensures that media and inducers diffuse quickly (about 30–60 s for a nanoscale particle).

#### PDMS Flow Cell

The flow cell was made using standard PDMS techniques. A silicon wafer mould was generated using optical lithography techniques at the Cornell Nanoscale Facility (Ithaca, NY). A 4 inch silicon wafer was coated with photoresist following a desired pattern. The wafer was then etched using a plasma machine. The length of the designed channel was set to 40 mm. Its width is 600 microns and height is 100 microns . Because of the extreme simplicity and the dimensions of the channel, the simpler SU8 lithography techniques would probably work as well. PDMS (mixed with curing agent, as described above) was then cast on top of the mould and baked at 80°C for 2 hours. Inlet and oulet were generated using blunt needles (Gauge 26). Unlike the usual PDMS flow chambers that are sealed to the glass using air plasma-induced covalent bonding, the flow chamber is not sealed so that it can be cleaned after the experiment and reused at will.

#### Assembling the device

A droplet of log-phase cells (10 microliters at about 2. 10^6^ cells/ml) was deposited on the coated coverslip. A clean wet membrane was put on top of it. In order for the membrane to be in contact with the PDMS, we waited about 15 minutes so that the water layer between membrane and coverslip mostly evaporates. The membrane then tends to naturally stick to the PDMS. However, excessive drying would tend to damage the cells and the membrane ultimately unsticks when completely dried out. The flow cell was then disposed on top of the membrane at the appropriate time, and a plastic plate was used to clamp the whole system. The flow cell was connected to tubing (tygon, GE) using Gauge 21 needles. Flows were driven by a peristaltic pump (Ismatec, IPC4) and media selection is controlled using an array of electric control valves (The Lee Company, LFAA1200118H) that were powered using a 12 volts power supply (MacMaster) and computer-controlled using a RS232 switch board (National Control Devices, PWM8x).

### Heating stage and objective heating devices

The microfluidic device was placed on a home made aluminium stage, mounted with two thermoelectric 72W devices (Melcor, CP 1.4-127-045L). Aluminium heatsinks (McMaster) were used to dissipate heat on the cold side of the modules. A powersupply with an integrated PID regulation (Oven Industries, 5C7-195) and temperature sensors (Oven Industries, TS-104 thermistors) were used to maintain the temperature at 32°C. Heating the objective as well (using a similar thermoelectric module and regulation) ensured that no heat was lost through the contact between the objective and the sample.

### Data Acquisition

The heating stage was put on the automated stage of an inverted microscope (Leica DMI6000B) with a Leica 63x N.A. 1.4 objective (HCX PL APO CS). This microscope was fully automated and computer controlled. A FireWire Hamamatsu camera (C4742-80-12AG) was used to acquire 12 bits pictures at 1344×1024 resolution. The camera was connected to a fast shutter (Uniblitz, VS35S2ZM1) using a TTL link to precisely control the synchrony with the electronic triggering of the camera. Thus we could use low exposure times (typically 200 ms). We wrote dedicated software in Matlab 7.2 to control every step of the experiment using a graphical user interface : image acquisition (using Matlab Image Acquisition Toolbox 2.10), microscope driving, temperature, pump and electrovalves control. Hardware (except the camera) were interfaced through RS232 ports. The source code is available upon request.

Images were acquired every 3 minutes. Autofocusing was achieved on phase contrast images by acquiring a stack of images and selecting the one with maximal gradient. Fluorescent images (of Venus, GFP, mCherry fluorophores) were acquired using the appropriate filter sets (Chroma, respectively : 41028, 41020, 31004) using 2×2 pixels binning. Using a narrow bandpass filter for GFP helped cutting out the naturally high cell autofluorescence in the green, thus improving the signal/noise ratio.

We noticed that the mean cell fluorescence levels tend to decrease with time (with a timescale of several hours) 7–8 hours after the beggining of the experiment. We found that a constant aeration of the media tanks (using an aquarium pump and bubblers from Aquatic Gardens) and large flow rates ( >30 µL/min ) prevented this phenomenon. Without the bubbling, the dissolved oxygen concentration in our medium reservoirs dropped to a very low level by the end of an overnight run. Oxygen is required for maturation of fluorescent proteins[27].

### Data analysis

Data were analyzed using a Matlab program. It was used to edit and correct cell contours generated by a segmenting program previously described[7]. The new capability to automatically track bud neck markers eased the monitoring of large cell colonies. Raw fluorescence images were retreated in order to substract the membrane autofluorescence contribution to the signal. See supplementary Text S1 for detail.

## Supporting Information

Figure S1 Overall principle of the setup. The whole setup is driven by a custom-made Matlab application run on a PC computer, through RS232 interface (except the camera which has FireWire interface). The microscope, the camera, the fluoresence shutter, the control unit for the thermo-electric devices (TE, stage and objective heaters) as well as for the electric valves can be remotely controlled. A TTL link between the fluorescence shutter and the camera ensures a better synchrony of these two devices.
(0.05 MB PDF)

Figure S2 Picture and sketch of the assembly of the setup. Picture and sketch of the assembly of the setup; Top: top view of the microfluidic device mounted on its stage. The whole flow cell ( coverslip+membrane+flow cell ) is clamped onto a heated aluminium alloy stage using screws attached to an aluminium top. Heating the stage as well as the objective using thermoelectric modules ensures a constant temperature.
(0.88 MB PDF)

Figure S3 Comparison of cell proliferation in a standard 2% agar setup versus the microfluidic sandwich setup. Cells carrying a copy of CDC10-YFP were grown in the microfluidic device for 12 hours in SCD. Timelapse of cell growth using the standard agar method was carried out as previously described[7]. On the left is shown a sketch of the respective setups. On the right are displayed a sequence of image taken during the experiment at indicated times. Each phase image is processed using custom software in order to identify cells and extract their contours. The very flat colonies in the microfluidic device allows one to image cells for 8–10 generations, versus about 5 using agar pads [7]. Enlargments on the right emphasize the difference between the two setups at high cell density.
(0.84 MB PDF)

Figure S4 Correlation of fitted γ and α with cell size (a-b) and cell-cycle phase (c-d), scored using the budneckmarker in Fig. 3. Numbers indicate the value of the coefficient of correlation. To determine what factors could affect the cell-to-cell variability of γ (which sets the peak height for a given τ in Fig. 2b), we correlated γ obtained for individual cells with cell size and cell cycle phase (using the red budneck marker MYO1-mCherry, which works similarly as CDC10-YFP). We did not see any obvious correlation (Fig. 10 a and b). The same analysis was done with α (Fig. 15c and d). We did not see any systematic fluorescence oscillations with the cell cycle for steady growth in 0xMet or 10xMet, in contrast to the G2 peak in expression from the Met regulon in rich medium[28].
(0.10 MB PDF)

Figure S5 Histograms for the G1/S trigger and the mitotic trigger. Histograms of timings associated to the various markers used in the pulsing experiments in Fig. 4. t0 , t1 , t2 and t3 are defined in the text (different for a and b).
(0.31 MB PDF)

Table S1 Timings of cell cycle events in triggering experimentsexperiments. WT Mother and Daughter represent control
(0.03 MB PDF)

Table S2 Strain and plasmid list
(0.04 MB PDF)

Text S1 Methods and models description
(2.65 MB DOC)

Movie S1 WT cell growth, cell contour and bud neck annotation. The movie corresponds to experiments shown in figure 1
(6.58 MB AVI)

Movie S2 Transient gene expression. 15 minutes MET3 induction upon media shift
(12.31 MB AVI)

Movie S3 The G1/S trigger. Repeated pulses of CLN2 (this movie corresponds to experiments displayed in Fig. 4c)
(14.09 MB AVI)

Movie S4 The mitotic exit trigger. Repeated pulse od CDC20 (this movie correspondes to experiments displayed in Fig. 4g).
(11.42 MB AVI)

## References

[1] Lord PG, Wheals AE (1981). Variability in individual cell cycles of sacchormyces cerevisiae.. J. Cell. Sci..

[2] Kron SJ, Styles CA, Fink GR (1994). Symmetric cell division in pseudohyphae of the yeast S*accharomyces Cerevisiae*.. Mol. Biol. Cell..

[3] Sveiczer A, Novak B, Mitchison JM (1996). The size control of fission yeast revisited.. J. Cell Sci..

[4] Huh WK, Falvo JV, Gerke LC, Carroll AS, Howson RW (2003). Global analysis of protein localization in budding yeast.. Nature.

[5] Nagai T, Ibata K, Park ES, Kubota M, Mikoshiba K (2002). A variant of yellow fluorescent protein with fast and efficient maturation for cell-biological applications.. Nat. Biotech..

[6] Shaner NC, Campbell RE, Steinbach PA, Giepmans BN, Palmer AE (2004). Improved monomeric red, orange and yellow fluorescent proteins derived from discosoma sp. red fluorescent protein.. Nat. Biotech..

[7] Bean JM, Siggia ED, Cross FR (2006). Coherence and timing of cell cycle start examined at single-cell resolution.. Mol. Cell..

[8] Gordon A, Colman-Lerner A, Chin TE, Benjamin KR, Yu RC (2007). Single-cell quantification of molecules and rates using open-source microscope-based cytometry.. Nat. Methods.

[9] Paliwal S, Iglesias PA, Campbell K, Hilioti Z, Groisman A (2007). MAPK-mediated bimodal gene expression and adaptive gradient sensing in yeast.. Nature.

[10] Cookson S, Ostroff N, Pang WL, Volfson D, Hasty J (2005). Monitoring dynamics of single-cell gene expression over multiple cell cycles.. Mol Syst Biol.

[11] Balaban NQ, Merrin J, Chait R, Kowalik L, Leibler S (2004). Bacterial persistence as a phenotypic switch.. Science.

[12] Smith AE, Zhang Z, Thomas CR, Moxham KE, Middelberg AP (2000). The mechanical properties of saccharomyces cerevisiae.. Proc Natl Acad Sci U S A..

[13] Sia SK, Whitesides GM (2003). Microfluidic devices fabricated in poly(dimethylsiloxane) for biological studies.. Electrophoresis.

[14] Burke D, Dawson D, Stearns T In *Methods in Yeast Genetics*, 2000.

[15] Mateus C, Avery S (2000). Destabilized green fluorescent protein for monitoring dynamic changes in yeast gene expression with flow cytometry.. Yeast.

[16] Lanker S, Valdivieso MH, Wittenberg C (1996). Rapid degradation of the G1 cyclin cln2 induced by cdk-dependent phosphorylation.. Science.

[17] Mumberg D, Muller R, Funk M (1994). Regulatable promoters of saccharomyces cerevisiae: comparison of transcriptional activity and their use for heterologous expression.. Nucl. Acid Res..

[18] Mao X, Hu Y, Liang C, Lu C (2002). Met3 promoter: A tightly regulated promoter and its application in construction of conditional lethal strain.. Curr. Microb..

[19] Di Talia S, Skotheim JM, Bean JM, Siggia ED, Cross FR (2007). The effects of molecular noise and size control on variability in the budding yeast cell cycle.. Nature.

[20] Amon A, Irniger S, Nasmyth K (1994). Closing the cell cycle circle in yeast: G2 cyclin proteolysis initiated at mitosis persists until the activation of G1 cyclins in the next cycle.. Cell.

[21] Costanzo M, Nishikawa JL, Tang X, Millman JS, Schub O (2004). Coherence and timing of cell cycle start examined at single-cell resolution.. Cell.

[22] de Bruin RA, McDonald WH, Kalashnikova TI, Yates J, Wittenberg C (2004). Cln3 activates G1-specific transcription via phosphorylation of the SBF bound repressor Whi5.. Cell.

[23] Johnston GC, Pringle JR, Hartwell LH (1977). Coordination of growth with cell division in the yeast saccharomyces cerevisiae.. Exp Cell Res..

[24] Louvion JF, Havaux-Copf B, Picard D (1993). Fusion of GAL4-VP16 to a steroid-binding domain provides a tool for gratuitous induction of galactose-responsive genes in yeast.. Gene.

[25] Cross FR, Siggia ED (2005). Mode locking the cell cycle.. PRE.

[26] Battogtokh D, Tyson JJ (2006). Periodic forcing of a mathematical model of the eukaryotic cell cycle.. PRE.

[27] Tsien RY (1998). The green fluorescent protein.. Annu Rev Biochem..

[28] Spellman PT, Sherlock G, Zhang MQ, Iyer VR, Anders K (1998). Comprehensive identification of cell cycle-regulated genes of the yeast S*accharomyces Cerevisiae* by microarray hybridization.. Mol. Biol. Cell.

