# Supplementary figures and images for: A Microfluidic Device for Temporally Controlled Gene Expression and Long-Term Fluorescent Imaging in Unperturbed Dividing Yeast Cells
Source: PLoS One. 2008 Jan 23;3(1):e1468. doi: 10.1371/journal.pone.0001468 (PMC2194624; doi:10.1371/journal.pone.0001468)

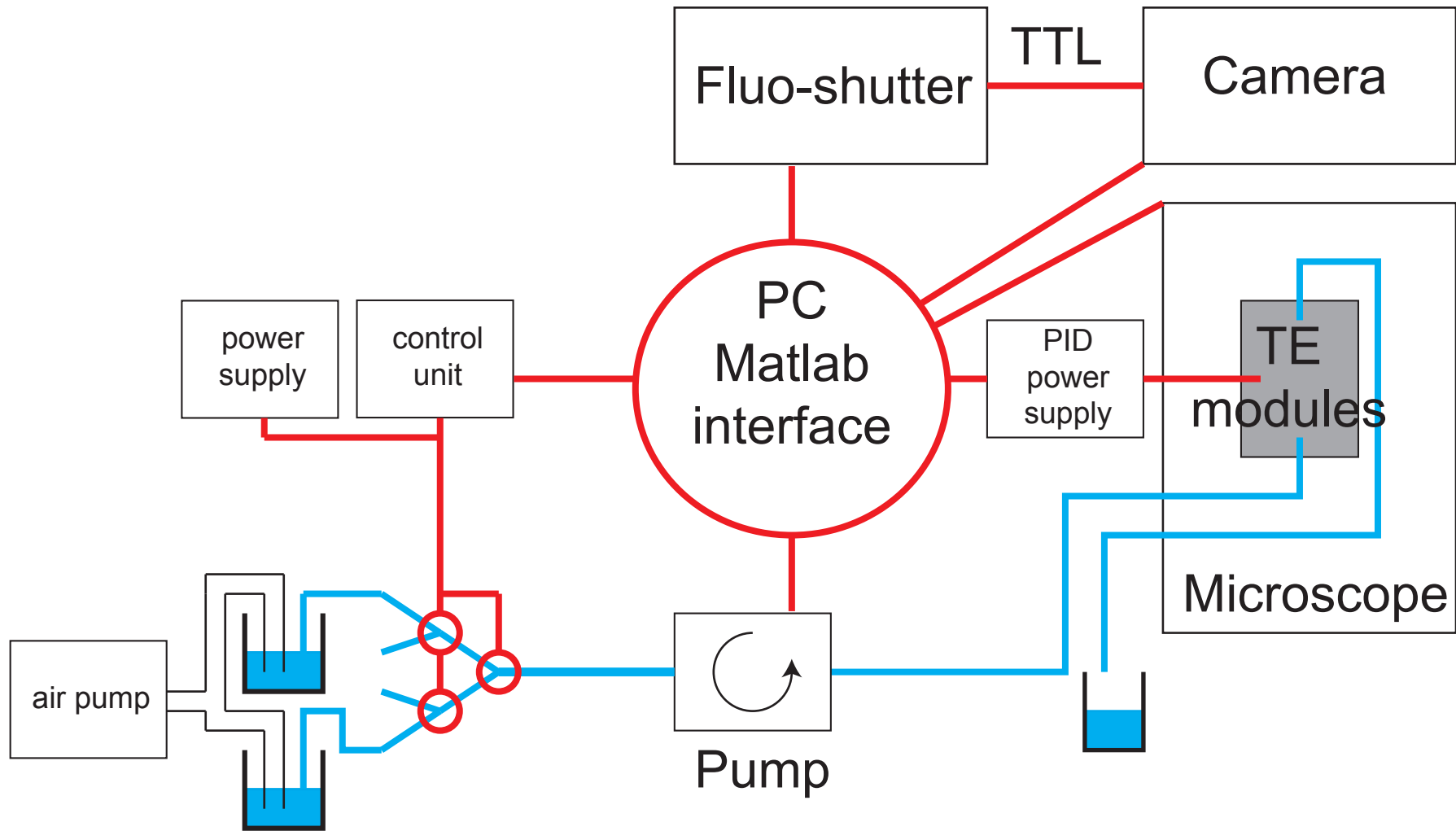

Supplement: Figure S1 — Overall principle of the setup. The whole setup is driven by a custom-made Matlab application run on a PC computer, through RS232 interface (except the camera which has FireWire interface). The microscope, the camera, the fluoresence shutter, the control unit for the thermo-electric devices (TE, stage and objective heaters) as well as for the electric valves can be remotely controlled. A TTL link between the fluorescence shutter and the camera ensures a better synchrony of these two devices. (0.05 MB PDF) [file pone.0001468.s001.pdf]

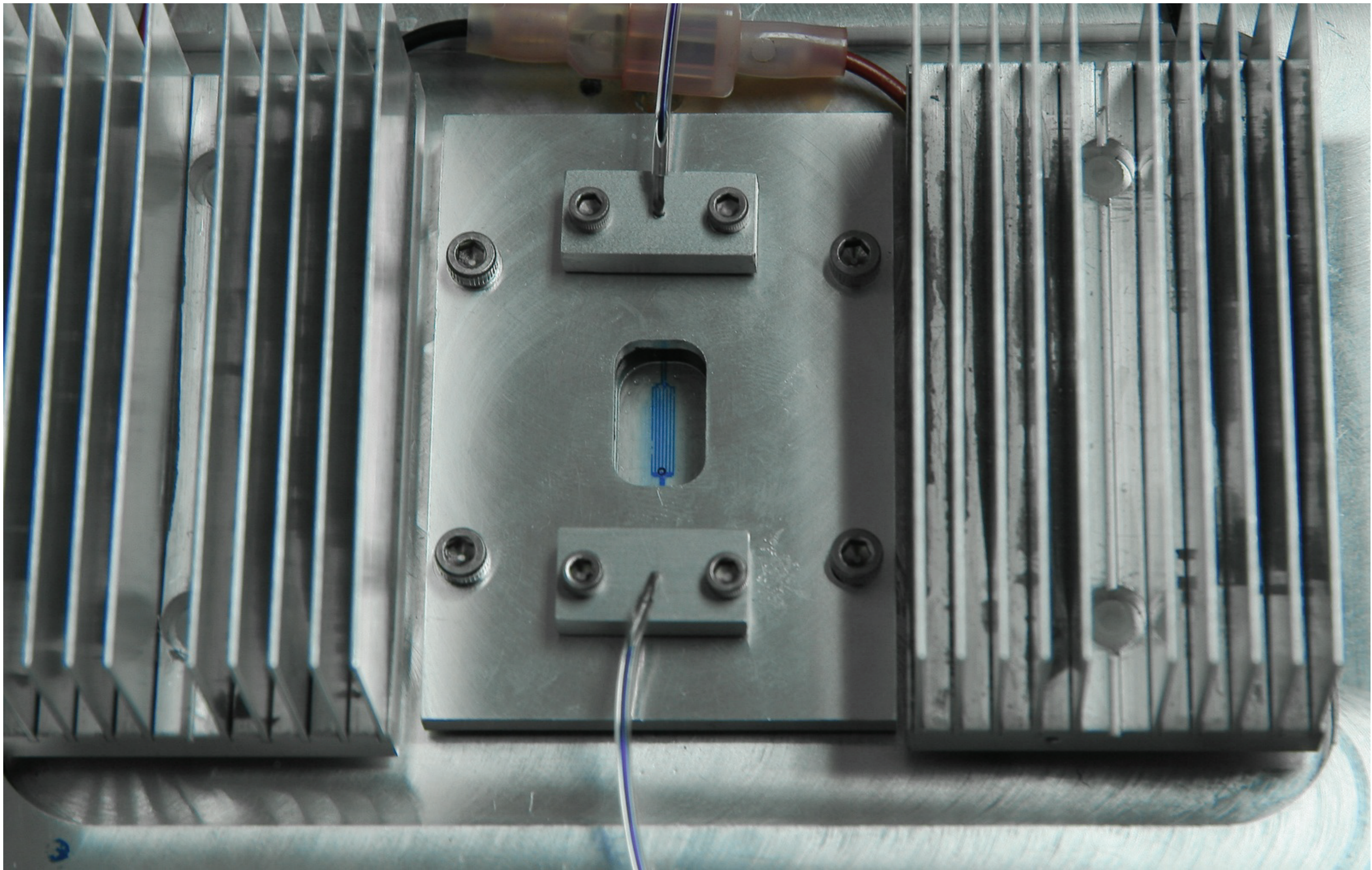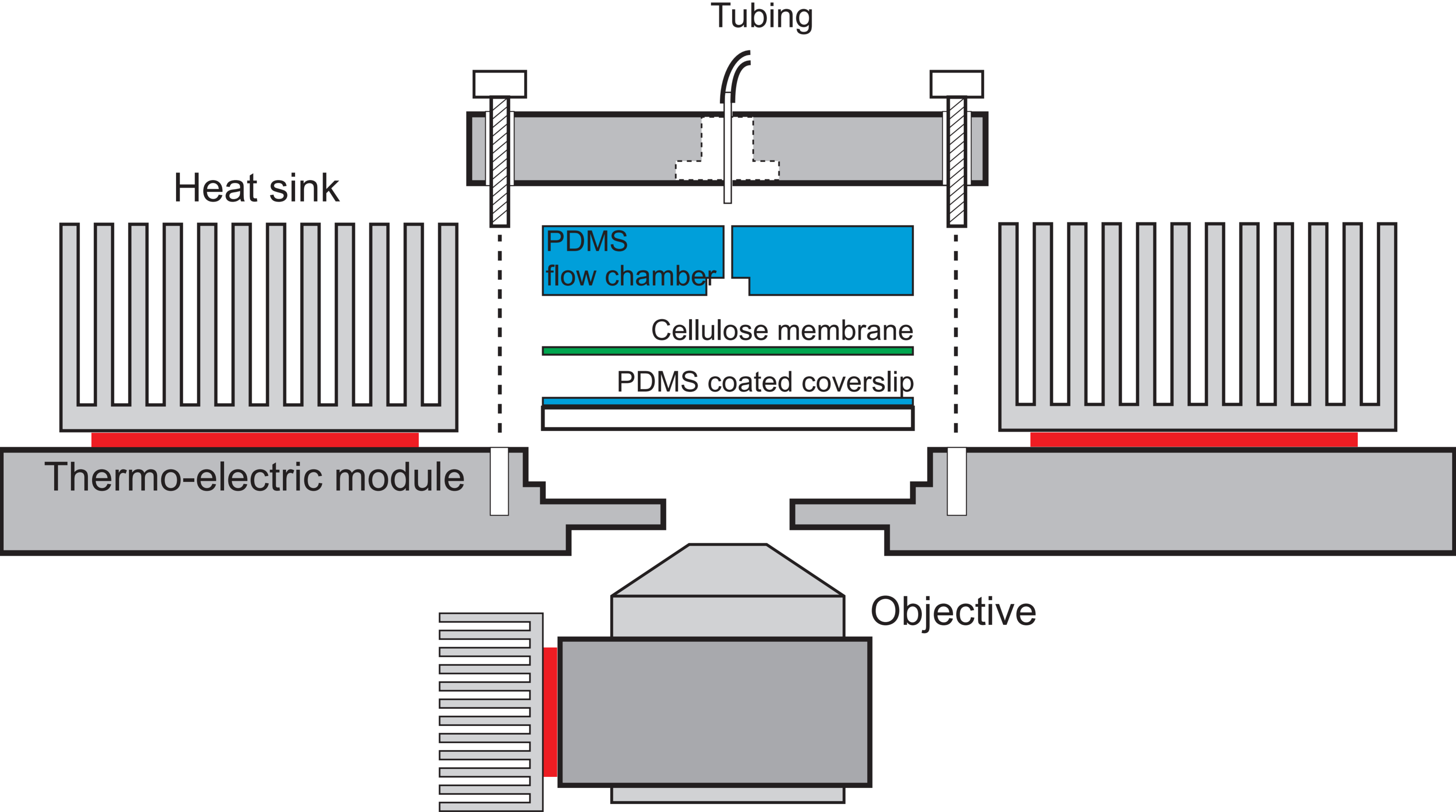

Supplement: Figure S2 — Picture and sketch of the assembly of the setup. Picture and sketch of the assembly of the setup; Top: top view of the microfluidic device mounted on its stage. The whole flow cell ( coverslip+membrane+flow cell ) is clamped onto a heated aluminium alloy stage using screws attached to an aluminium top. Heating the stage as well as the objective using thermoelectric modules ensures a constant temperature. (0.88 MB PDF) [file pone.0001468.s002.pdf]

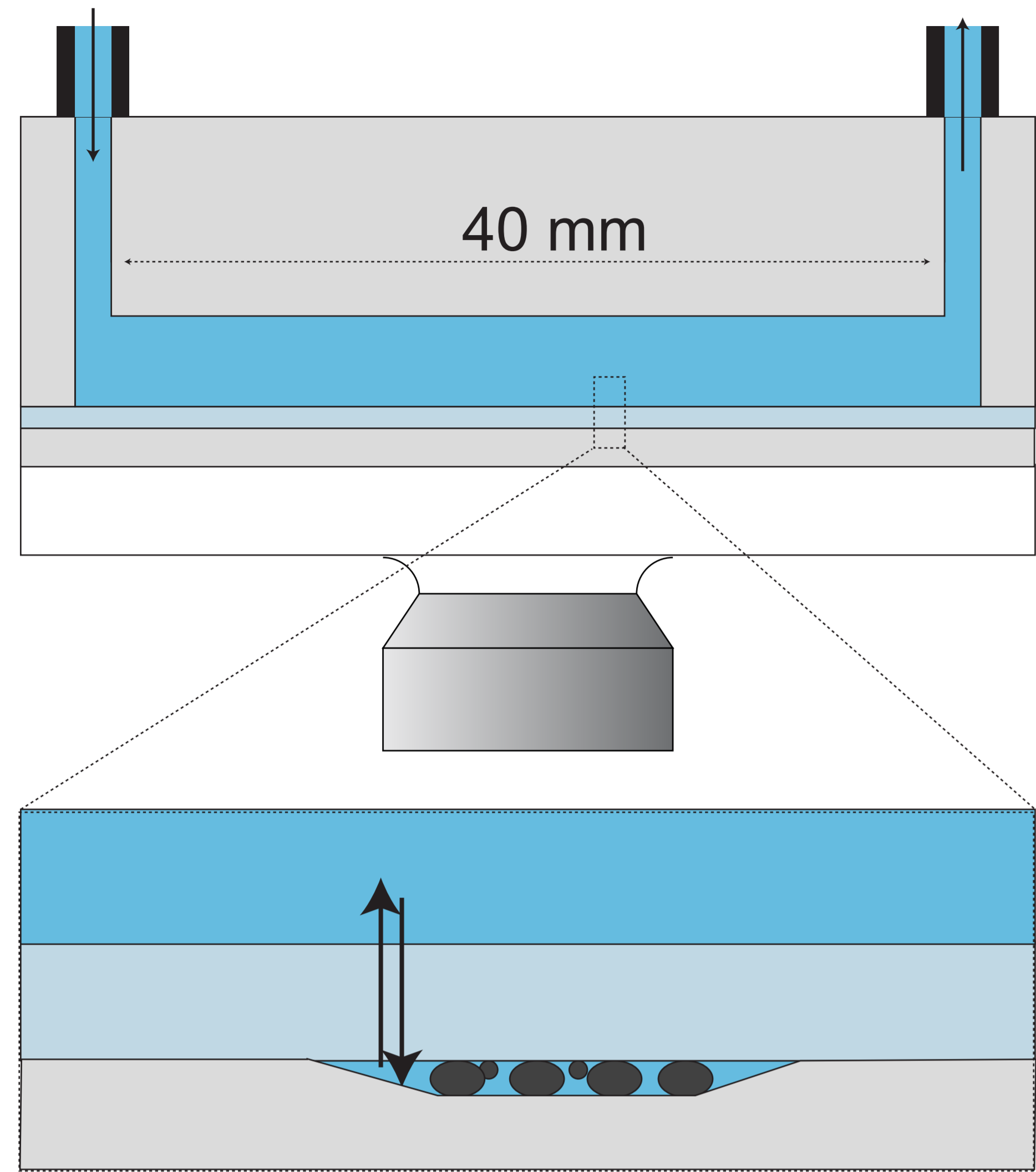

Flow chamber

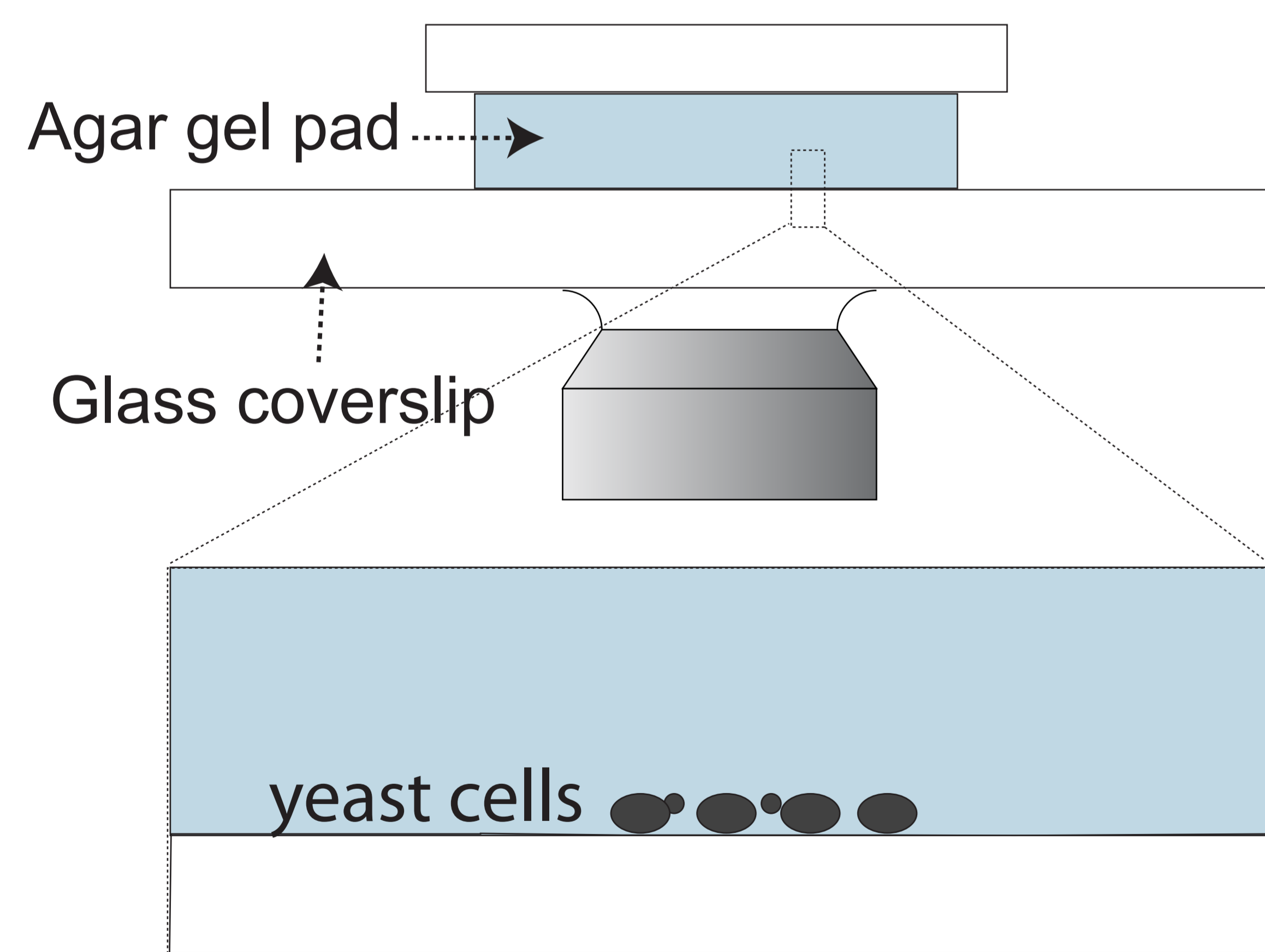

Agar pad setup

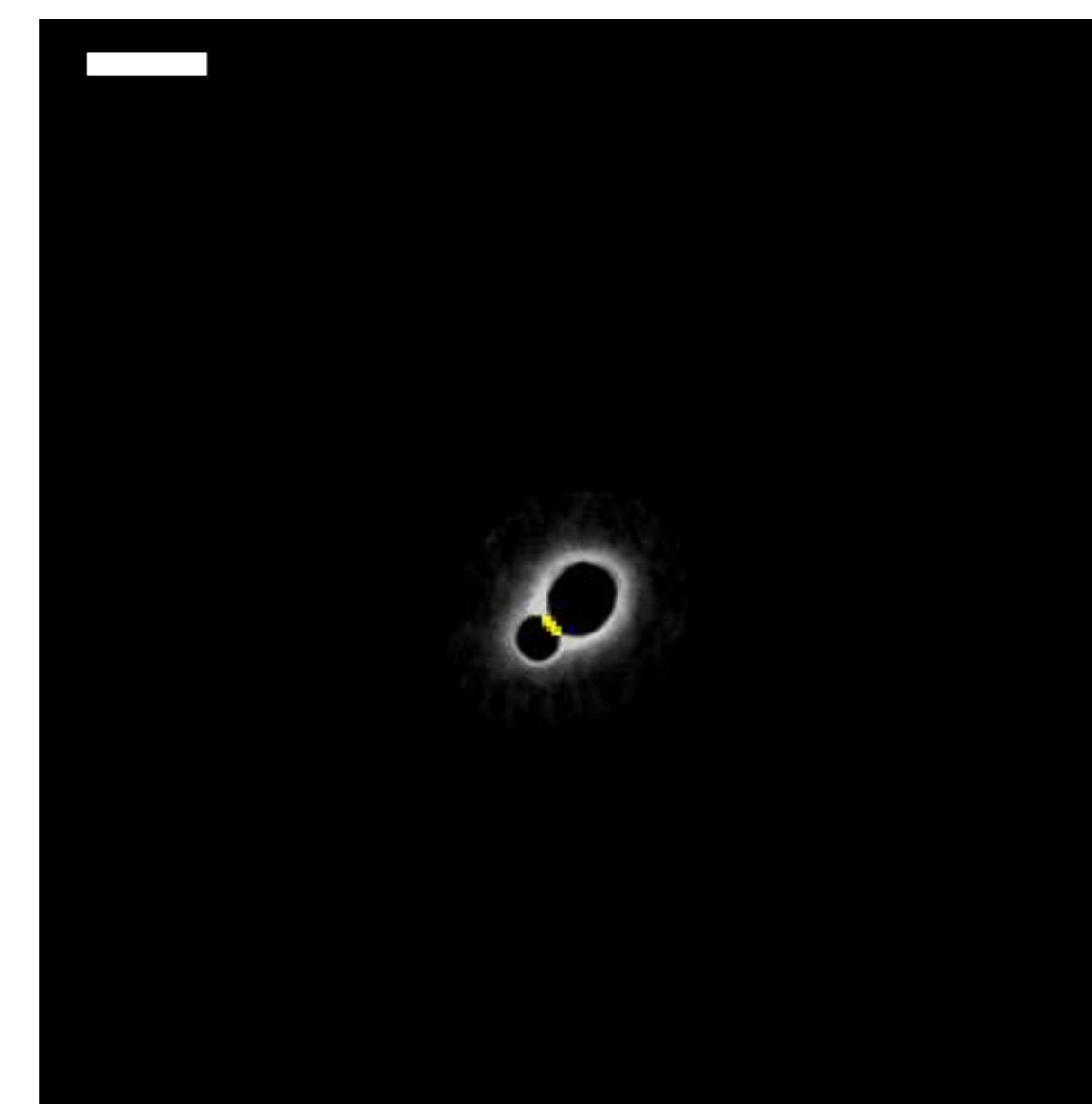

t=0 min

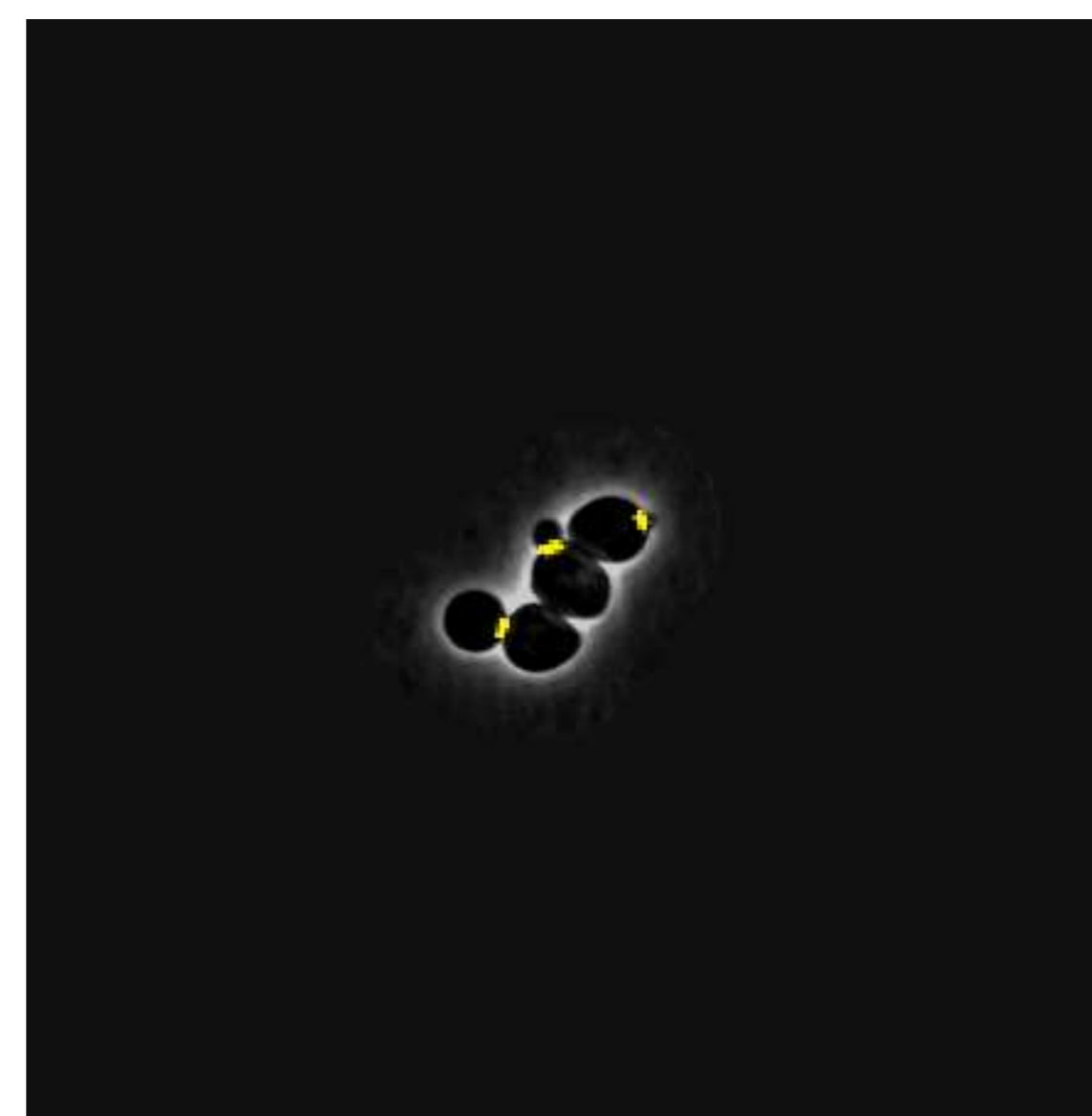

t=150 min

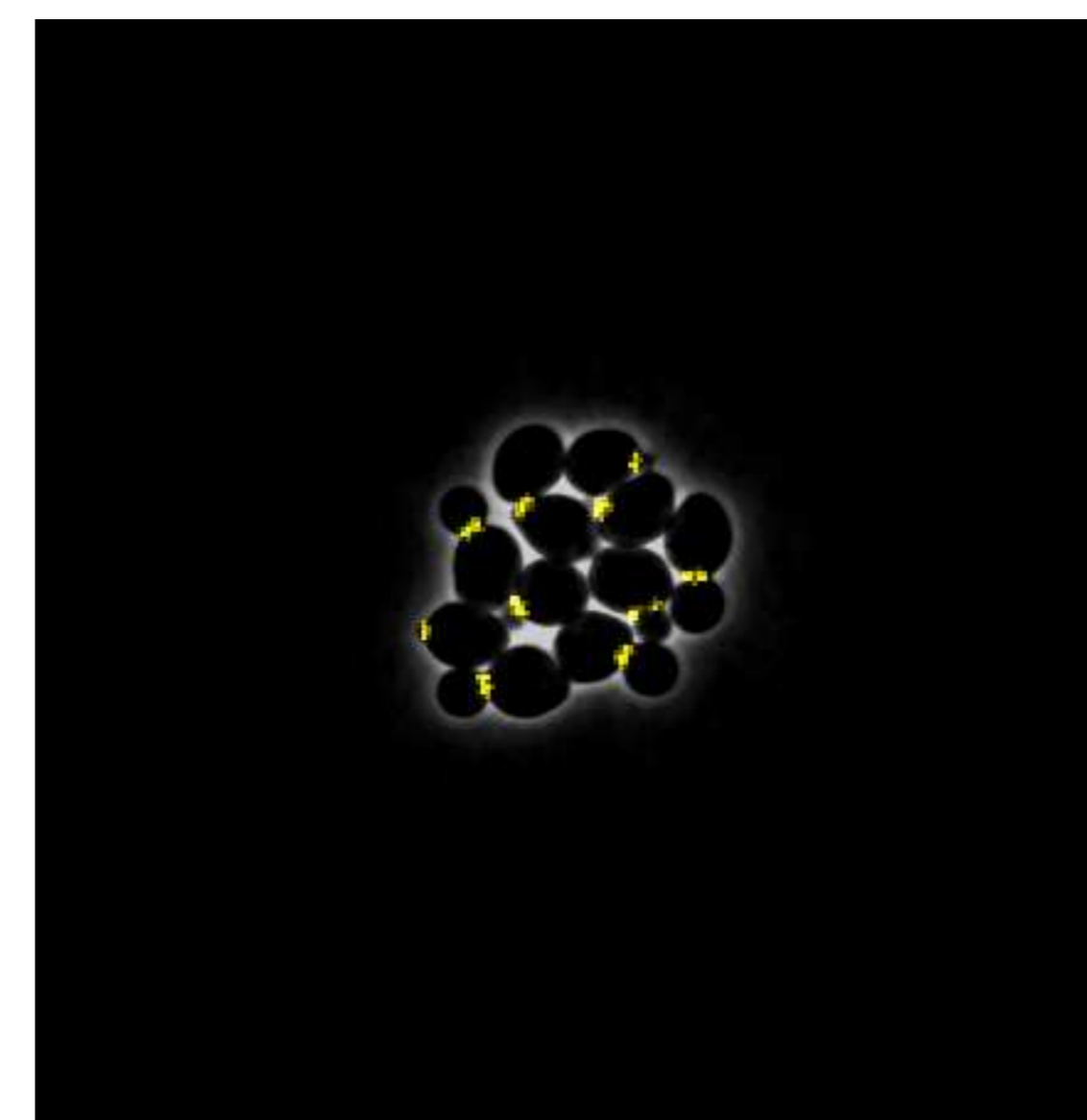

t=300 min

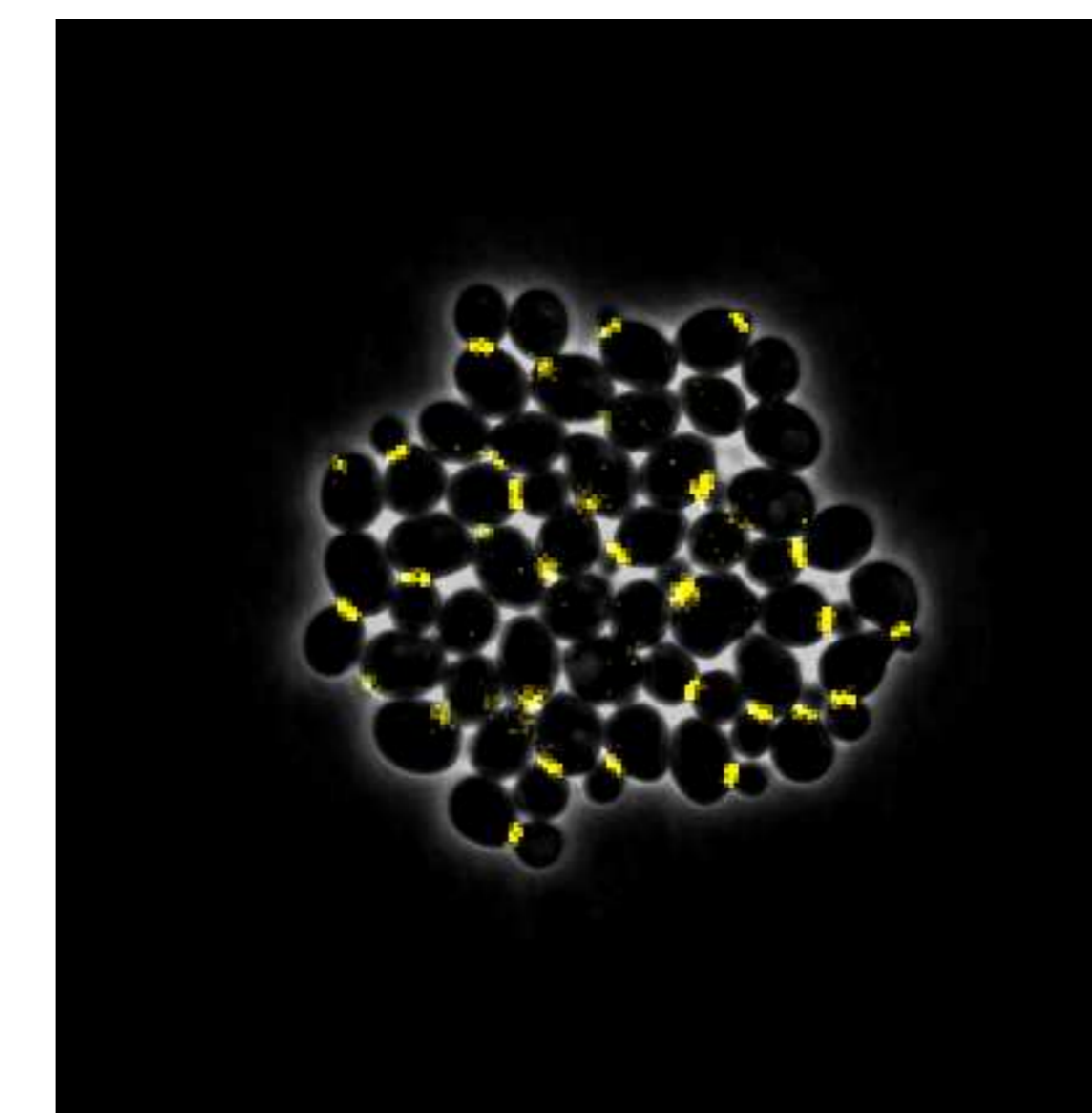

t=450 min

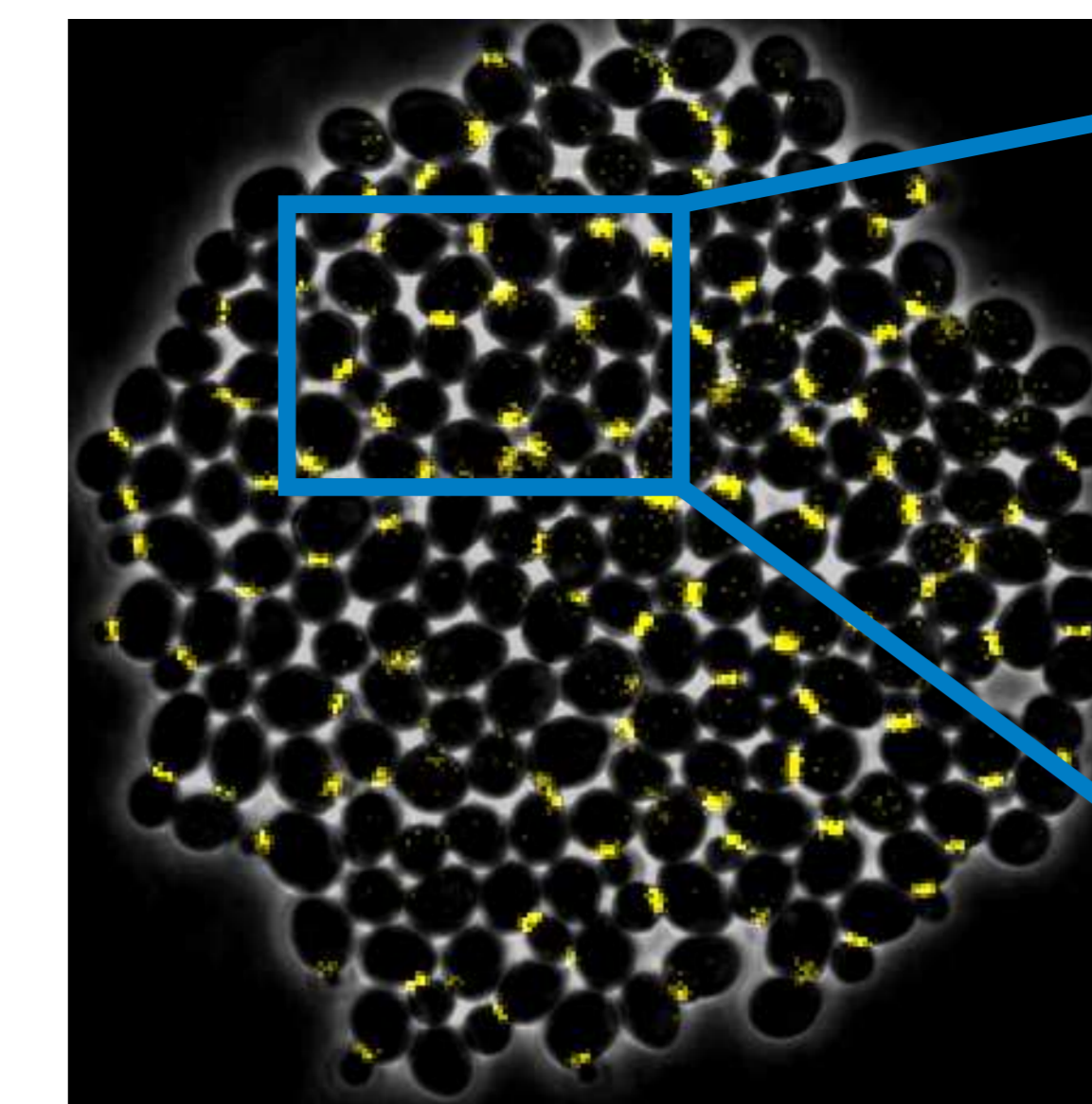

t=600 min

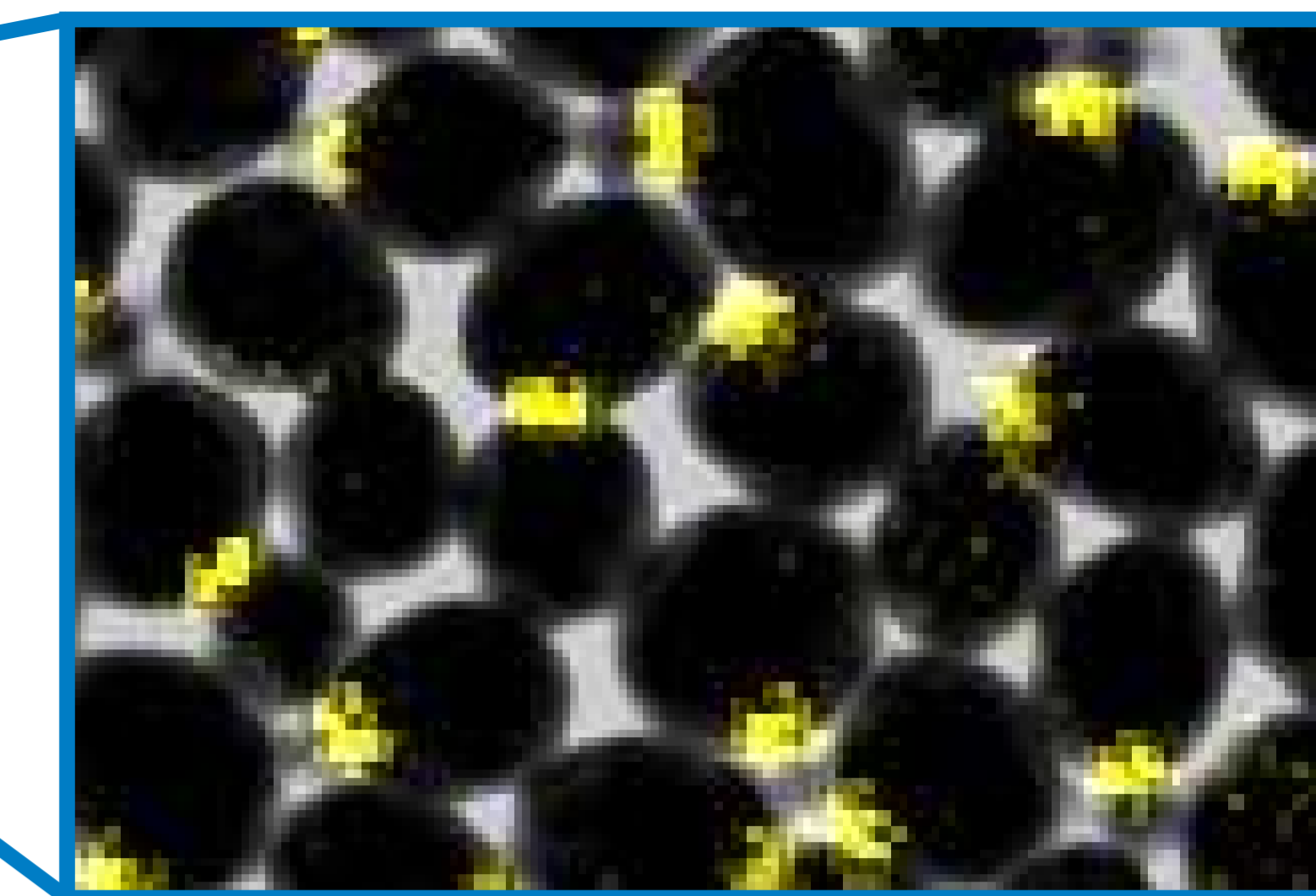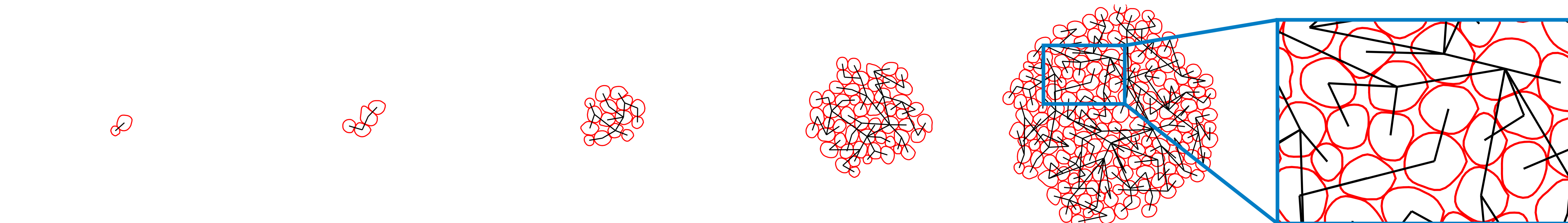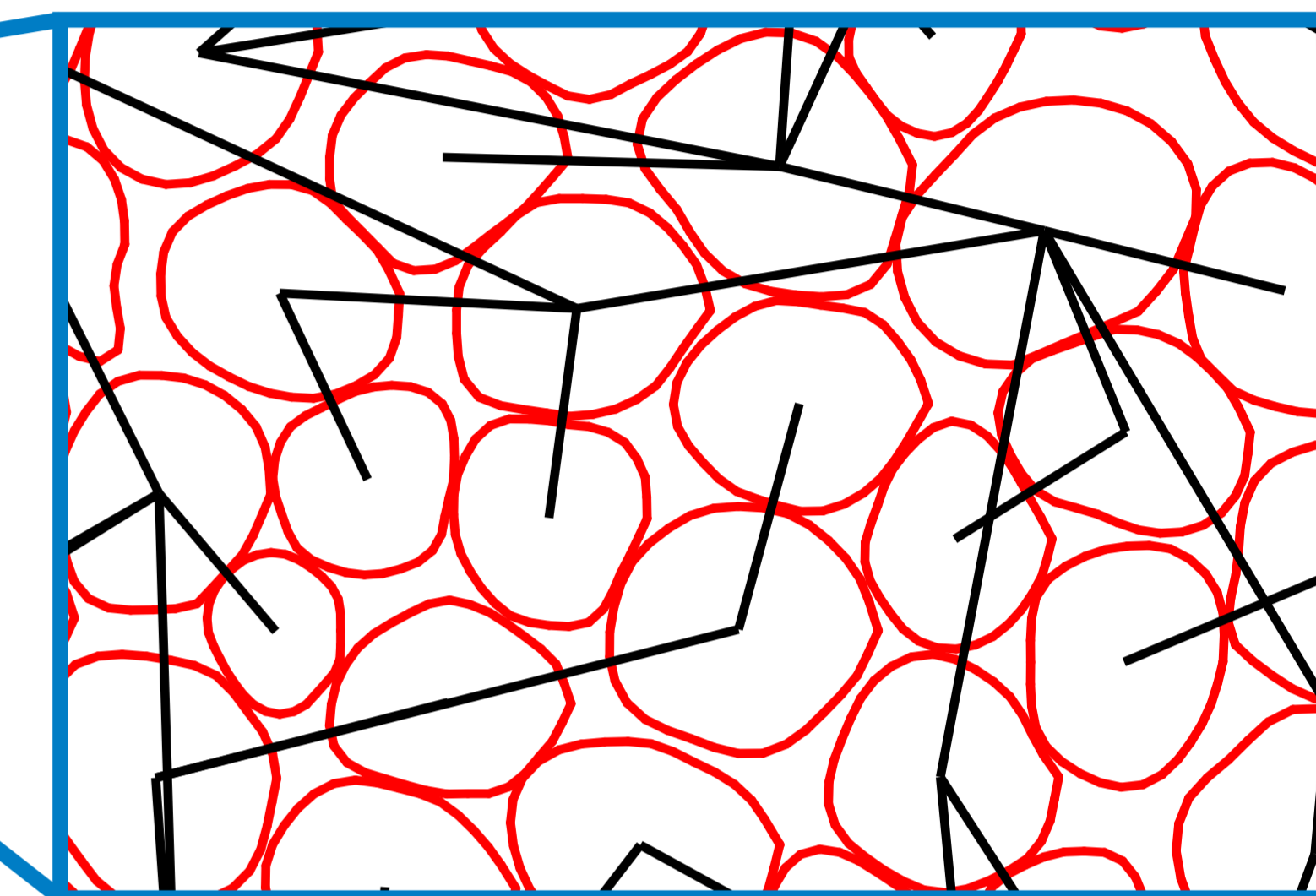

Supplement: Figure S3 — Comparison of cell proliferation in a standard 2% agar setup versus the microfluidic sandwich setup. Cells carrying a copy of CDC10-YFP were grown in the microfluidic device for 12 hours in SCD. Timelapse of cell growth using the standard agar method was carried out as previously described[7]. On the left is shown a sketch of the respective setups. On the right are displayed a sequence of image taken during the experiment at indicated times. Each phase image is processed using custom software in order to identify cells and extract their contours. The very flat colonies in the microfluidic device allows one to image cells for 8–10 generations, versus about 5 using agar pads [7]. Enlargments on the right emphasize the difference between the two setups at high cell density. (0.84 MB PDF) [file pone.0001468.s003.pdf]

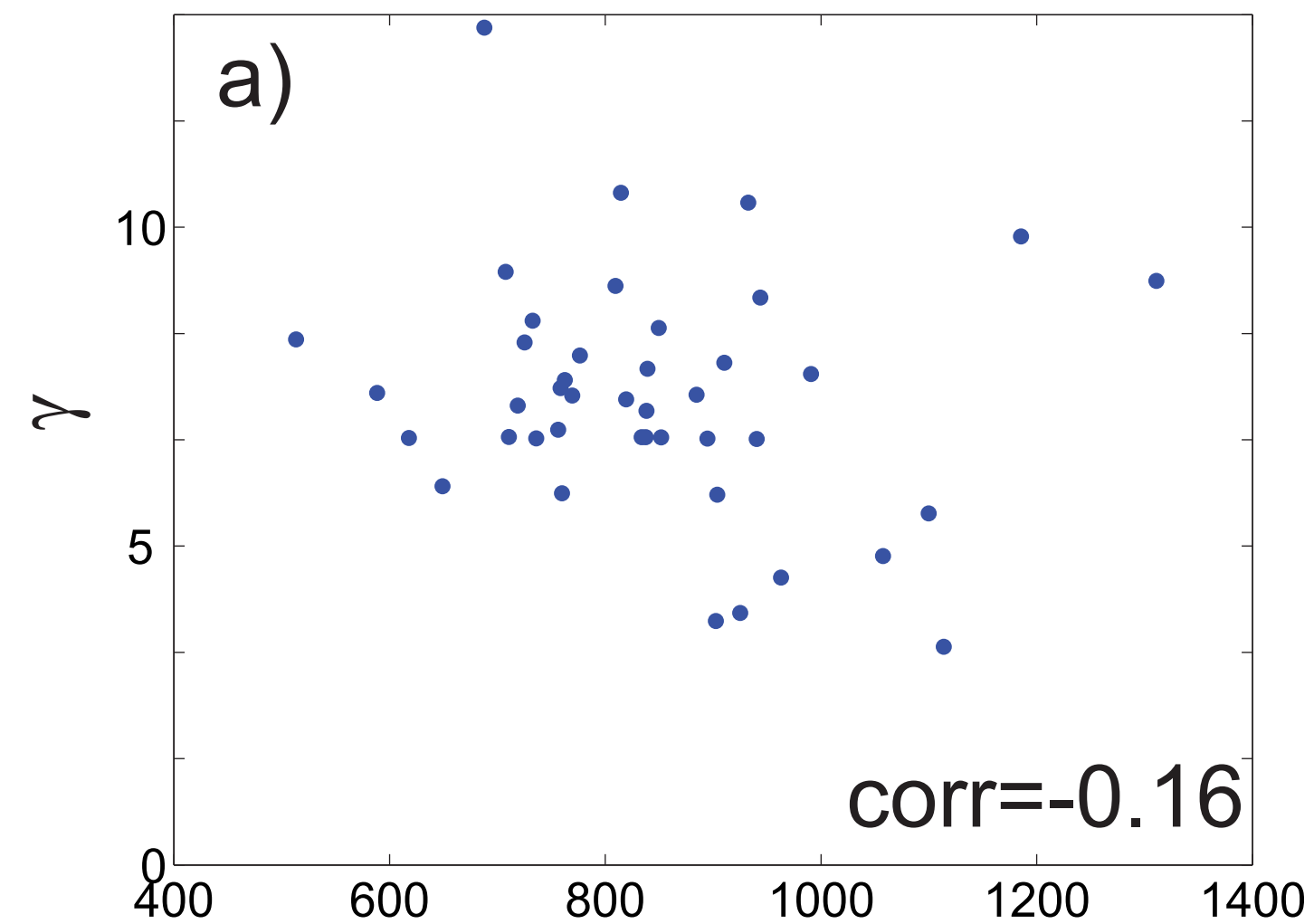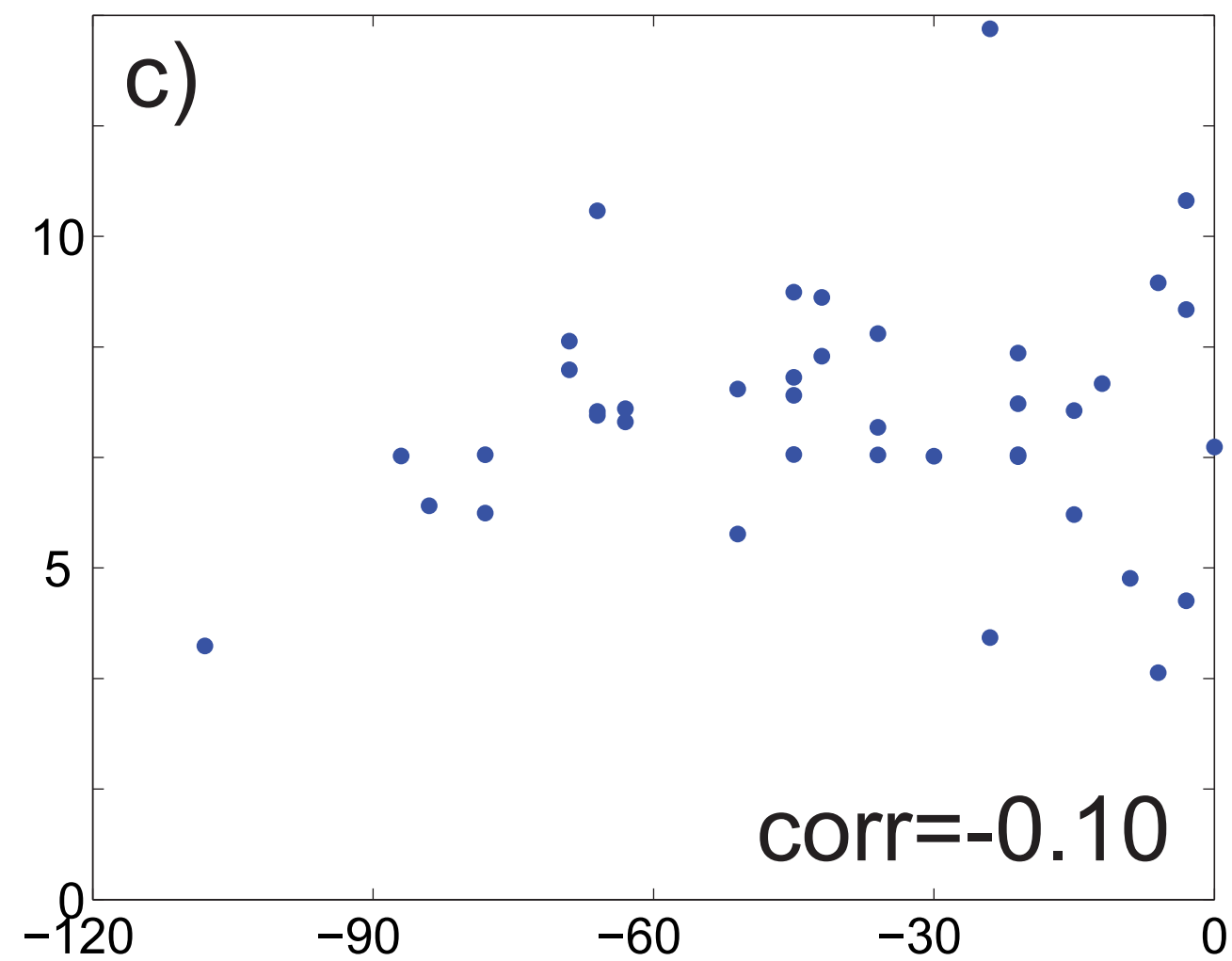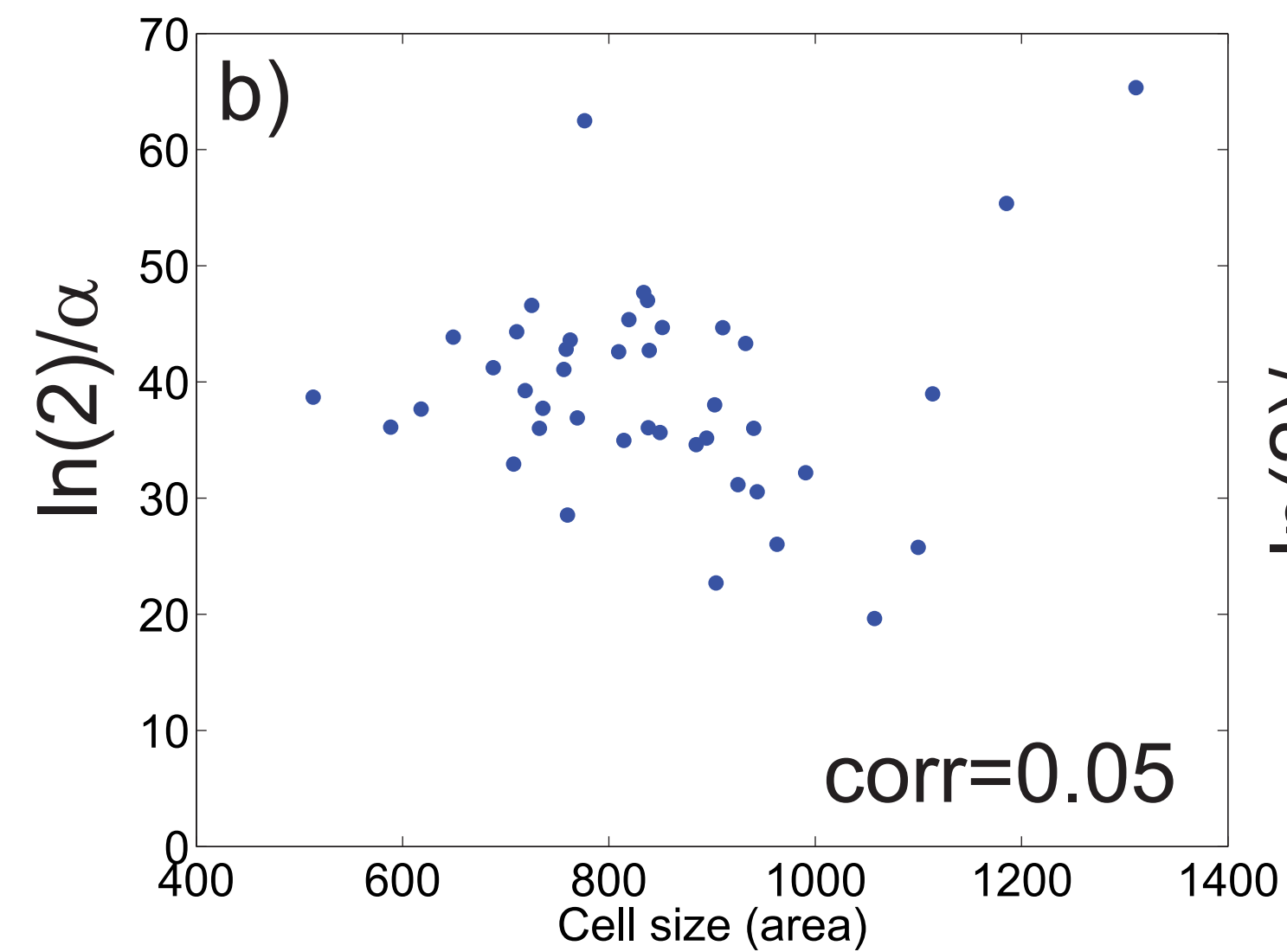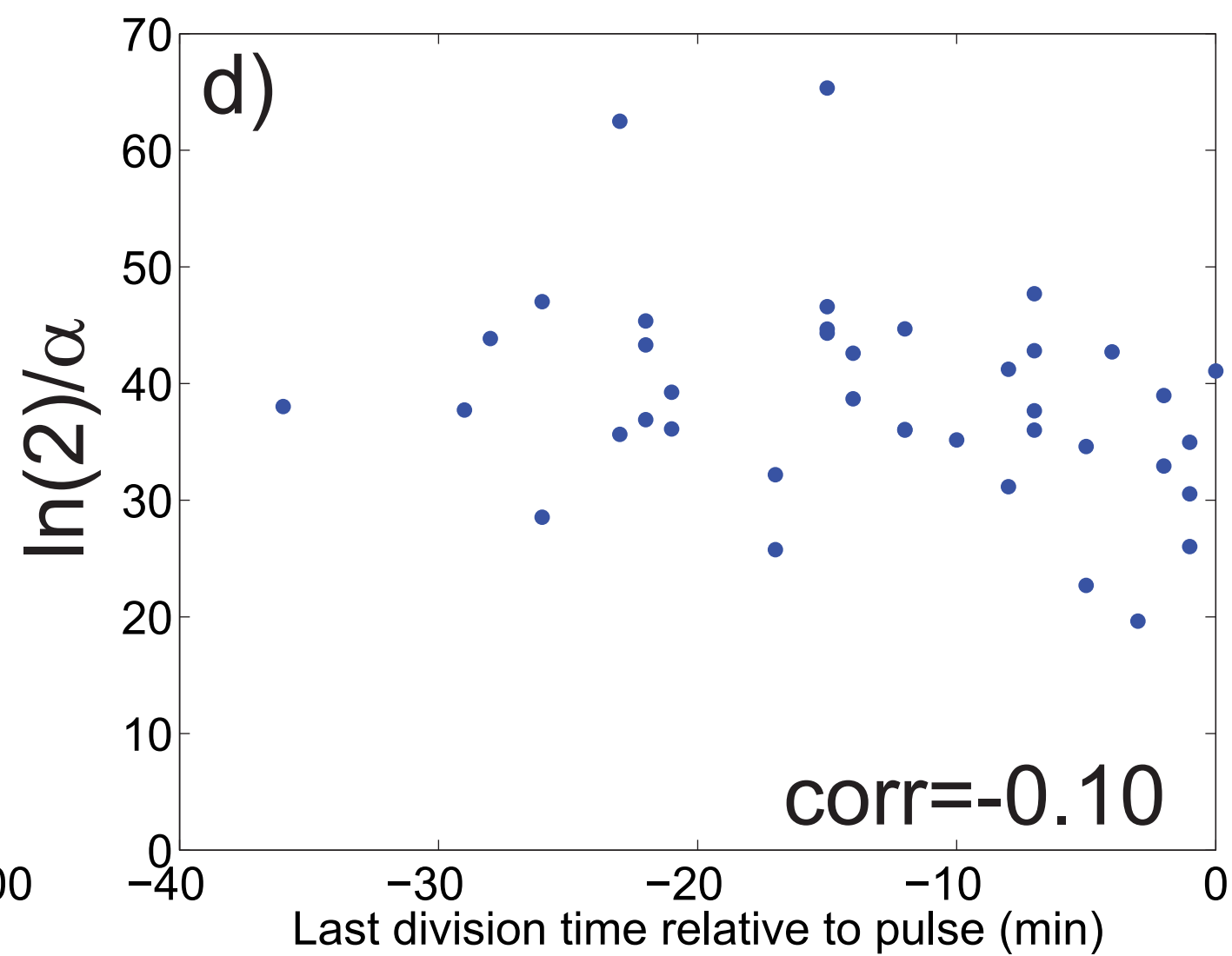

Supplement: Figure S4 — Correlation of fitted γ and α with cell size (a-b) and cell-cycle phase (c-d), scored using the budneckmarker in Fig. 3. Numbers indicate the value of the coefficient of correlation. To determine what factors could affect the cell-to-cell variability of γ (which sets the peak height for a given τ in Fig. 2b), we correlated γ obtained for individual cells with cell size and cell cycle phase (using the red budneck marker MYO1-mCherry, which works similarly as CDC10-YFP). We did not see any obvious correlation (Fig. 10 a and b). The same analysis was done with α (Fig. 15c and d). We did not see any systematic fluorescence oscillations with the cell cycle for steady growth in 0xMet or 10xMet, in contrast to the G2 peak in expression from the Met regulon in rich medium[28]. (0.10 MB PDF) [file pone.0001468.s004.pdf]

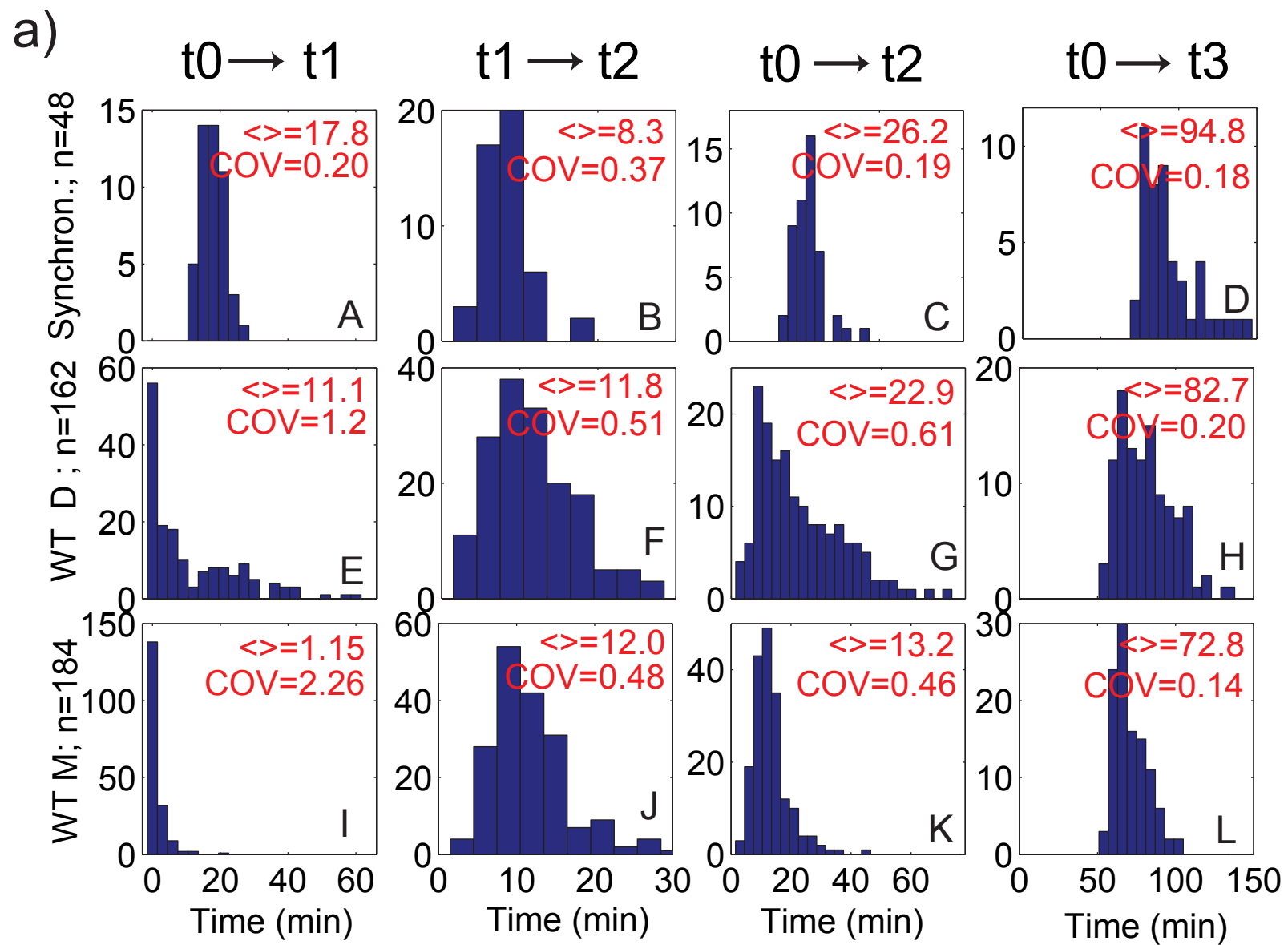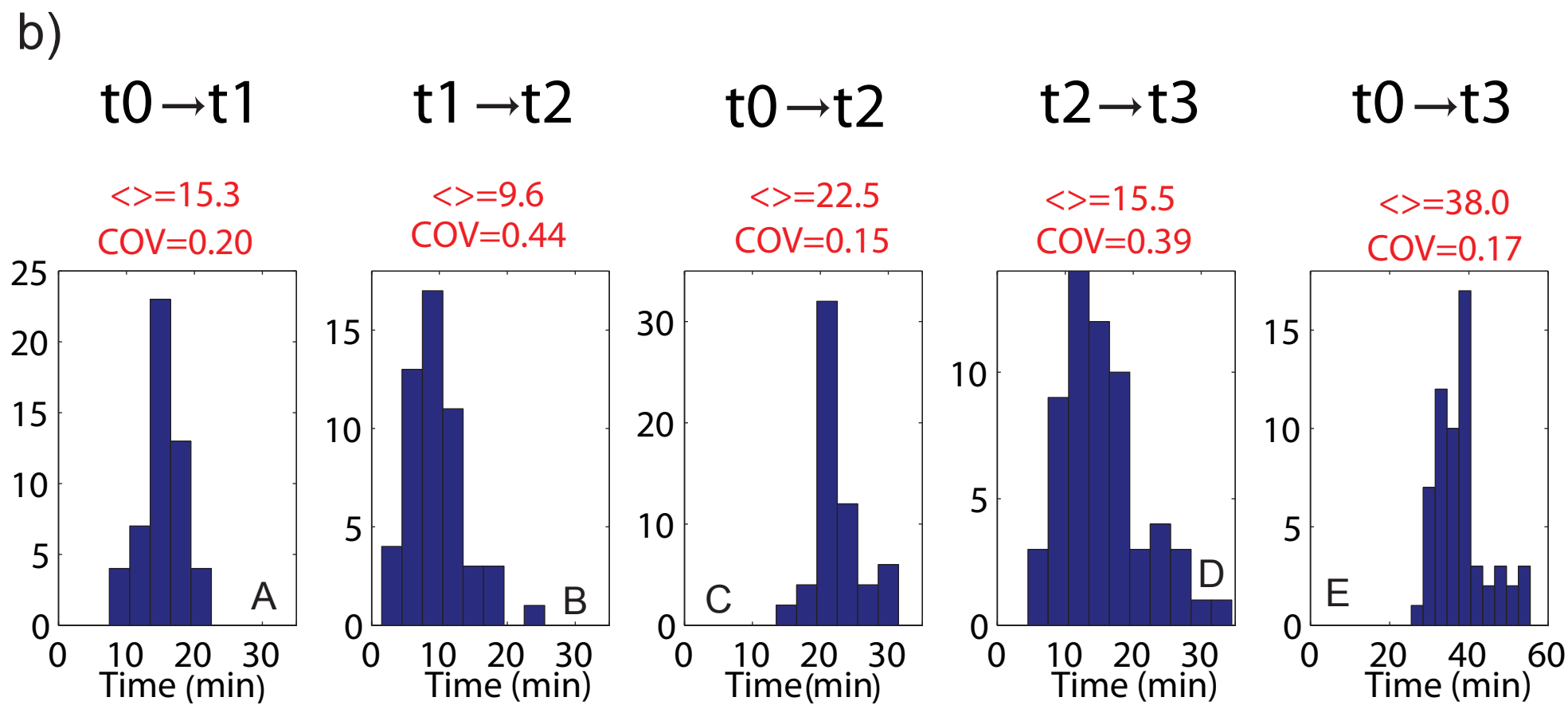

Supplement: Figure S5 — Histograms for the G1/S trigger and the mitotic trigger. Histograms of timings associated to the various markers used in the pulsing experiments in Fig. 4. t0 , t1 , t2 and t3 are defined in the text (different for a and b). (0.31 MB PDF) [file pone.0001468.s005.pdf]
