# Supplementary material for: A Microfluidic Device for Temporally Controlled Gene Expression and Long-Term Fluorescent Imaging in Unperturbed Dividing Yeast Cells
Source: PLoS One. 2008 Jan 23;3(1):e1468. doi: 10.1371/journal.pone.0001468 (PMC2194624; doi:10.1371/journal.pone.0001468)
Supplement: Table S1 — Timings of cell cycle events in triggering experimentsexperiments. WT Mother and Daughter represent control (0.03 MB PDF) [file pone.0001468.s006.pdf]

Experiment	From	To	Mean (min)	COV
WT Mother	Division (t_0)	*WHI5* exit (t_1)	1.15 ± 0.19	2.26
WT Mother	*WHI5* exit (t_1	Budding (t_2)	12.0 ± 0.4	0.48
WT Mother	Division (t_0)	Budding (t_2)	13.2 ± 0.4	0.46
WT Mother	Division (t_0)	Division (t_3)	72.8 ± 1.0	0.14
WT Daughter	Division (t_0)	*WHI5* exit (t_1)	11.1 ± 1.0	1.2
WT Daughter	*WHI5* exit (t_1)	Budding (t_2)	11.8 ± 0.5	0.5
WT Daughter	Division (t_0)	Budding (t_2)	22.9 ± 1.0	0.6
WT Daughter	Division (t_2)	Division (t_3)	82.7 ± 1.6	0.2
G1/S trigger	Trigger (t_0)	*WHI5* exit (t_1)	17.8 ± 2.5	0.2
G1/S trigger	*WHI5* exit (t_1)	Budding (t_2)	8.3 ± 0.4	0.37
G1/S trigger	Trigger (t_0)	Budding (t_2)	26.6 ± 0.7	0.19
G1/S trigger	Trigger (t_0)	Division (t_3)	94.8 ± 2.5	0.18
Mitotic trigger	Trigger (t_0)	Anaphase (t_1)	15.3 ± 0.42	0.2
Mitotic trigger	Anaphase (t_1)	Division (t_2)	9.6 ± 0.58	0.44
Mitotic trigger	Trigger (t_0)	Division (t_2)	22.5 ± 0.44	0.15
Mitotic trigger	Trigger (t_2)	Budding (t_3)	15.5 ± 0.78	0.39
Mitotic trigger	Trigger (t_0)	Budding (t_3)	38.0 ± 0.83	0.17

Table 1: Timings of cell cycle events in triggering experiments. WT Mother and Daughter represent control experiments
