# Supplementary material for: A Microfluidic Device for Temporally Controlled Gene Expression and Long-Term Fluorescent Imaging in Unperturbed Dividing Yeast Cells
Source: PLoS One. 2008 Jan 23;3(1):e1468. doi: 10.1371/journal.pone.0001468 (PMC2194624; doi:10.1371/journal.pone.0001468)
Supplement: Table S2 — Strain and plasmid list (0.04 MB PDF) [file pone.0001468.s007.pdf]

Strains (all W303)	Genotype	Origin
GC103-05B	MAT α *CDC10*-YFP-LEU2 -KanMx	this study, from [7]
GC87-03	MAT a *MYO1*::*MYO1*-mCherry::SpHis5 *GAL1*::*GAL1*pr-Venus-deg-TRP1 ADE2	this study
GC116-01	MAT a *MYO1*::*MYO1*-mCherry-SpHis5 TRP1:*MET3*pr-Venus-deg ADE2	this study
GC118-02	MAT a *MYO1*::*MYO1*-Venus-SpHis5 TRP1:*MET3*pr-Venus-deg ADE2	this study
OW01-12C	MAT a ADE2::GALL-*CDC20* *CDC20*::LEU2 HIS3::TUB1-GFP *CDC10*-YFP-LEU2	this study
GC60-06	MAT α TRP1::*GAL1*-yEVenus-deg ADE2	this study
GC53-01B	MAT a *MYO1*::*MYO1*-mCherry-SpHis5 *CLN2*::*CLN2*pr-Venus-deg ADE2	this study
GC36-03C	MAT α TRP1::TRP1-*MET3*pr-*CLN2* cln1::HIS3 *CLN2*Δ *cln3*::LEU2 -GFP-KanMX LEU2::*CDC10*-YFP ade2	this study
**Plasmids**	**Features**	**Origin**
pKT355	mCherry-ADH-SpHi5 tagging plasmid	Bi Lab (U. Penn.)
pJB04T	*ACT1*pr-yEGFP-deg TRP1	pSVA17 [7]
pGC04D	*ACT1*pr-yEVenus-deg TRP1	this study
pGC08D	*CLN2*pr-yEVenus-deg TRP1	this study
pGC25D	*MET3*pr-yEVenus-deg TRP1	this study
pRS426-*MET3*-HA-Hog1	*MET3*pr-HA-Hog1 URA3	Engelberg Lab
pYL29	*GAL1*pr-yEVenus-deg TRP1	this study
**Primer sequence**	**Sequence**	**Template**
MYO1 tagging 5'	AAATATTGATAGTAACAATGCACAGAGTAAAATTTTCAGTATACACATACGATTTAGGAGA	pKT355
MYO1 tagging 3'	GGATATAAAGTCTTCCAAATTTTTAAAAAAAAGTTCGTTACTGGAGGCGTTAGTATC	pKT355
*MET3*pr 5'	ATGTggatccTTTAGTACTAACAGAGAC	
*MET3*pr 3'	cGcGttaattaaTGTTAATTATACTTTATTCTT	
*GAL1*pr 5'	ATGCTAGCggatccAGCTCTAGTACGGATTAGAAGC	p405*GAL1*[17]
*GAL1*pr 3'	AGTCATCGttaattaaCTAGAATCCGGGGTTTTTTCTCC	p405*GAL1*[17]
	lower case indicate restriction sites	
