# Supplementary material for: A Microfluidic Device for Temporally Controlled Gene Expression and Long-Term Fluorescent Imaging in Unperturbed Dividing Yeast Cells
Source: PLoS One. 2008 Jan 23;3(1):e1468. doi: 10.1371/journal.pone.0001468 (PMC2194624; doi:10.1371/journal.pone.0001468)
Supplement: Text S1 — Methods and models description (2.65 MB DOC) [file pone.0001468.s008.doc]

Supplementary material : Methods and Models

G.Charvin, F.R. Cross, E.D. Siggia

gcharvin@rockefeller.edu

The Rockefeller University, 1230 York Avenue, New York, USA

Contents

Data acquisition and analysis using custom MATLAB software 2

Segmentation of cell contours from phase contrast images 2

Segmentation of bud neck using fluorescent bud neck markers 3

Frame by frame annotation procedure 5

Background subtraction in raw fluorescence images 7

Screenshots 9

Model for the pulses of gene expression 11

Measurement of Venus maturation time using protein translation block 11

Induction of Venus-deg upon a pulse of inducer 12

Fluorescence concentration and dilution effect 12

DATA ACQUISITION AND ANALYSIS USING CUSTOM MATLAB SOFTWARE

Segmentation of cell contours from phase contrast images

Our segmentation code is designed for phase images (see Fig. T1a below), and is based on morphological transformations and thresholding, with only secondary use of edge detection. Phase contrast images can have multiple halos that give rise to fragmented and nested edges and in dense cell clusters the useful information is the small bright pockets between cells rather than outlines of the cells themselves. A DIC image has more 'information' than phase, but a lot of internal cell structure is also visible that is superfluous for cell segmentation. Using a whole cell fluorescent marker for segmentation would allow one fewer gene reporters, and a phase image requires negligible light by comparison with fluorescence, minimizing photo-damage.

Italic function names below refer to the MATLAB Image Processing Toolbox. The first step is a series of dilation-erode operations (*imclose*) on a coarse scale, comparable to a cell to define a mask around the cell cluster. The next step is a flood fill operation (*imfill*), which raises the level of each connected dark patch until it reaches a saddle point in the intensity. Thus portions of cells will be filled in by constant intensity plateau, which are identified as peaks in an intensity histogram (12 bit images are essential at this stage). When there are vacuoles the intensity plateau can be very fragmented as in Fig. T1b . Nevertheless the plateau allow us to define for each image global intensity levels for thresholding. Bright regions are non-cell, dark regions, within the cell cluster mask are cells. This gives us a binary cell body image (Fig. T1d).

The cell body image can be refined using edge information. Standard Canny edge detection is done after the flood-fill operation and each continuous edge is fit to splines and divided into arcs with a curvature of one sign. Edges with too few pixels or with too large a radius of curvature (white edges in Fig. T1c) are discarded. For each arc, an implied center is found and an implicit inside/outside defined. If the intensity inside is larger than that outside (e.g. the red arcs in Fig. T1c) the contour is discarded; it is a phase contrast oscillation. Contours bounding a low intensity region (green in Fig. T1c) define together with their centers (green dots), a wedge that is assumed to be cell interior and is added to the cell body image. The magenta contours in Fig. T1c are assumed to be inter-cellular holes and an attempt is made to close them and then define those areas as non-cell.

We then compute the distance from each cell body pixel in Fig. T1d to the nearest boundary. We compute plausible saddle points of this function using a smoothed second derivative matrix plus an explicit test on the symmetry of the intensity image in a circle around the putative saddle. Nearby

saddles are merged. Then a cut is made from the saddle to the two closest boundaries. This routine though tedious, is much more accurate than watershed segmentation which would introduce many spurious cuts in Fig. T1d. Of course vacuoles lead to inappropriate cuts, but at this stage we have no means to distinguish a vacuole from inter-cellular space as is apparent in Fig. T1e.

Finally a automated clean up operation is done, to remove fragments that are too small, or very elongated (see Fig. T1f) and edges are defined from the binary image and overlaid on the phase image in Fig. T1g.

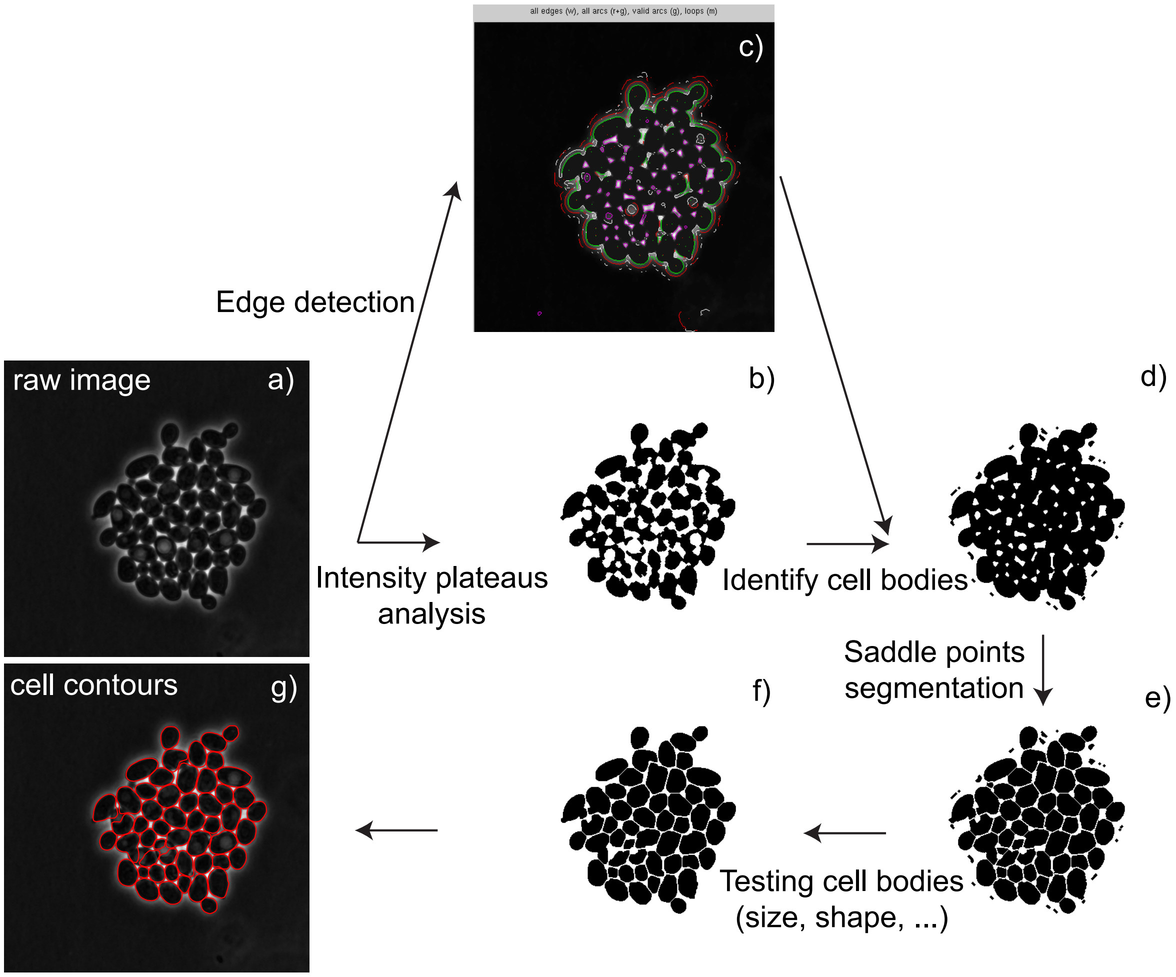

Figure T1: Steps of the segmentation routine used to retrieve cell contours.

Segmentation of bud neck using fluorescent bud neck markers

Bud necks fluorescent markers (such as CDC10-YFP or MYO1-mCherry) were used to score cell budding and division. Bud necks were detected in the appropriate fluorescent image by intensity

thresholding. Pixel clusters above threshold were labelled. Small (< 30 pixels) clusters were discarded.

Frame by frame annotation procedure

In order to track cell contour and bud necks across time points, we wrote a dedicated graphical user interface in MATLAB (celltracker4, see screenshot on figure T5) that allows for frame-by-frame visualization and annotation of the movie. The procedure was the following (see figure T2 a-d) : raw cell contours (obtained from the segmentation procedure, see above) at frame n+1 were mapped onto the contours of frame n. The mapping routine identifies which cell contour i at frame n+1 has the maximal overlap with contour j at frame n. Most of the cell contours are succesfully mapped using this procedure (see magenta contours on figure T2c). If no corresponding cell is found on frame n+1, we consider that the segmentation was not able to detect this cell correctly and add the contour of frame n to frame n+1 (lost cells, blue contour on Figure T2c). In addition, successfully mapped cell contours are submitted to various further tests : if the new cell contour is too small, if its center is too far from the one on previous frame, or has some prononced concavity, the new contour is discarded and replaced by the corresponding contour on frame n (blue contour on Figure T2c) . New buds are displayed in yellow. When they appear, they need to be manually connected to their mother cell in order to build the pedigree tree. If needed, the use of budneck markers makes this assignment unambiguous.

The frame to frame mapping procedure typically takes a fraction of a second to a few seconds depending on the cell cluster size. Once it is done, manual correction can be made to improve each contour. Usually, about 5% of the cells have segmentation defects, most of which are automatically fixed by using the corresponding contour at the previous frame. About the same fraction of sucessfully mapped cells may need corrections to improve contour fidelity. Typically, it takes about 30s of human attention to annotate (mark parentage, redraw contours) a field of 20 cells. The annotation procedure is usually iterated from the beginning to the end of the movie. Exponential growth obviously makes the process slow down with time. However, it is possible to systematically discard new cells so that the number of tracked contours stays constant (see Figure 3 in the manuscript).

Once all the cell contours are annotated, the same mapping procedure is repeated with bud neck markers (see Figure T2e-h). New budnecks are assigned to a cell body and are recorded as a budding event. We again map budnecks from time n+1 to n and color code by category. Lost bud neck contours can mean either cell cytokinesis occurred (in which case the contour has to be removed by the user) or could be a problem with tracking. This has to be evaluated by the user. Color coding the budneck mapping between times is essential in a large field of cells since it is impossible to mentally overlay the images and focus on those budneck markers that change.

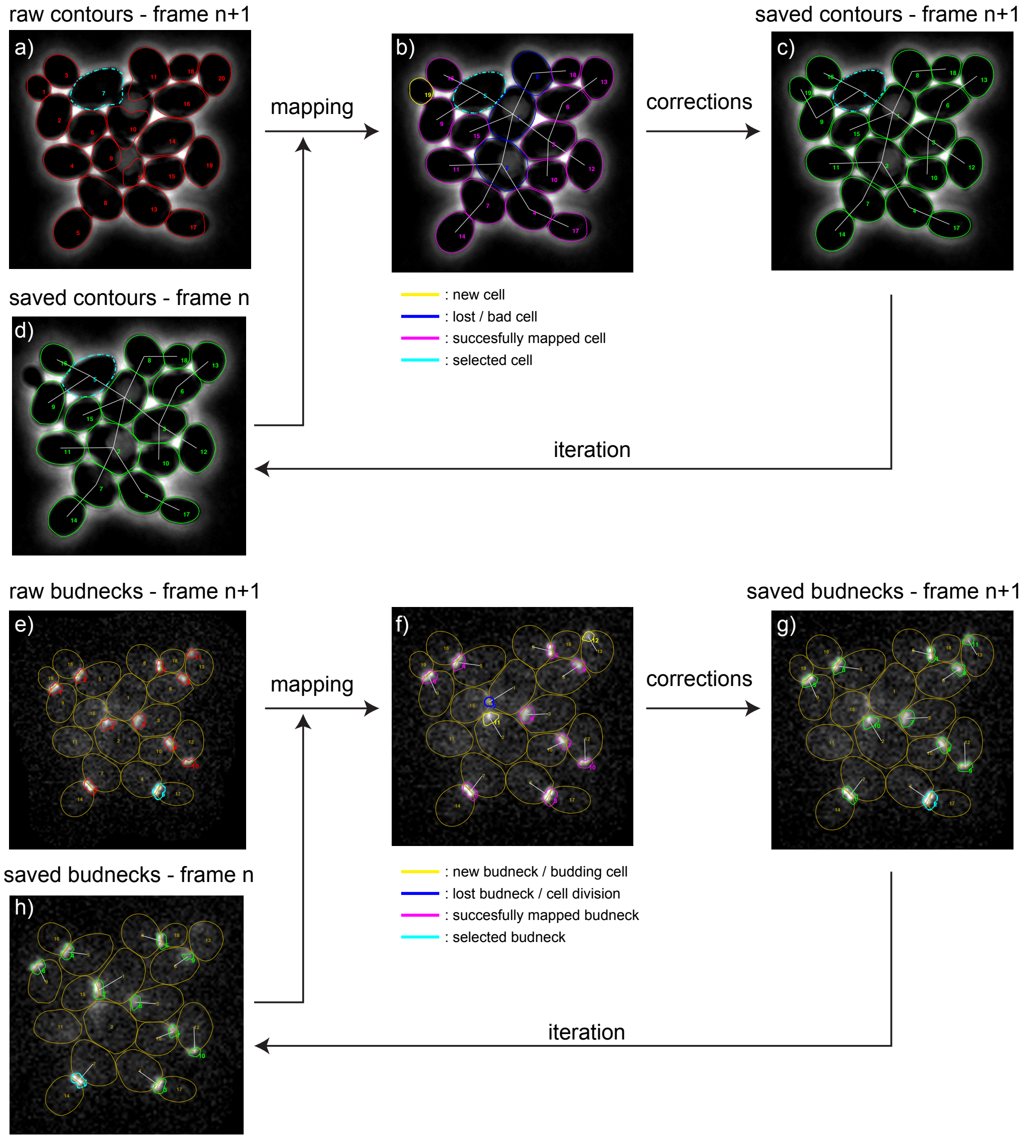

Figure T2: Sketch of the procedure used to track cell contour and bud necks across time points (frames). Top set of images is for cell contours, bottom is for bud necks.

Background subtraction in raw fluorescence images

The dialysis membrane we are using to confine the cells is somewhat autofluorescent and exaggerates the inhomogeneities of illumination across the field of view. The background was systematically slightly brighter in the center of the field of view compared to the edges. In addition, the background slowly increased with time, so that pixel intensities of the signal of interest (a fluorescent marker) drift. The reason for this increase in unclear. In order to fix these issues, we used the following image treatment to consistently subtract the background in all frames of the movie : frame 1 was used to evaluate the background profile across the image, since only a small fraction of the field of view is covered by cells. The cell cluster was discarded and the image was morphologically opened using a structuring element and then interpolated (filled) to get an estimate of the intensity profile in the absence of cells, i.e the background (see the red line at the bottom left on figure T3, and compare to the black line, which corresponds to the raw image). The background image was then subtracted from the raw image at frame 1 (see "subtracted" image and blue line on left panel of figure T3) which compensates for the inhomogenous illumination.

To infer the background profile at later times, we considered the pixels outside of the cell cluster and found the multiplicative factor which best mapped those pixels to their values at frame 1. The same factor was then used to scale the complete background image which was then subtracted from frame n (see middle and right panel on figure T3, corresponding to n=120 and n=200).

This procedure was globally efficient at keeping constitutive reporter signal in the same range of intensity across the movie. It was especially useful for a dim signal (like WHI5-GFP) or in the red channel, where inhomegeneities are more prononced. On the other hand, the substraction procedure introduces more noise in the image. In addition, it can not deal with variations of the background that are particular to large cell clusters such as the fluorescence halo visible for frame 200 in Figure T3.

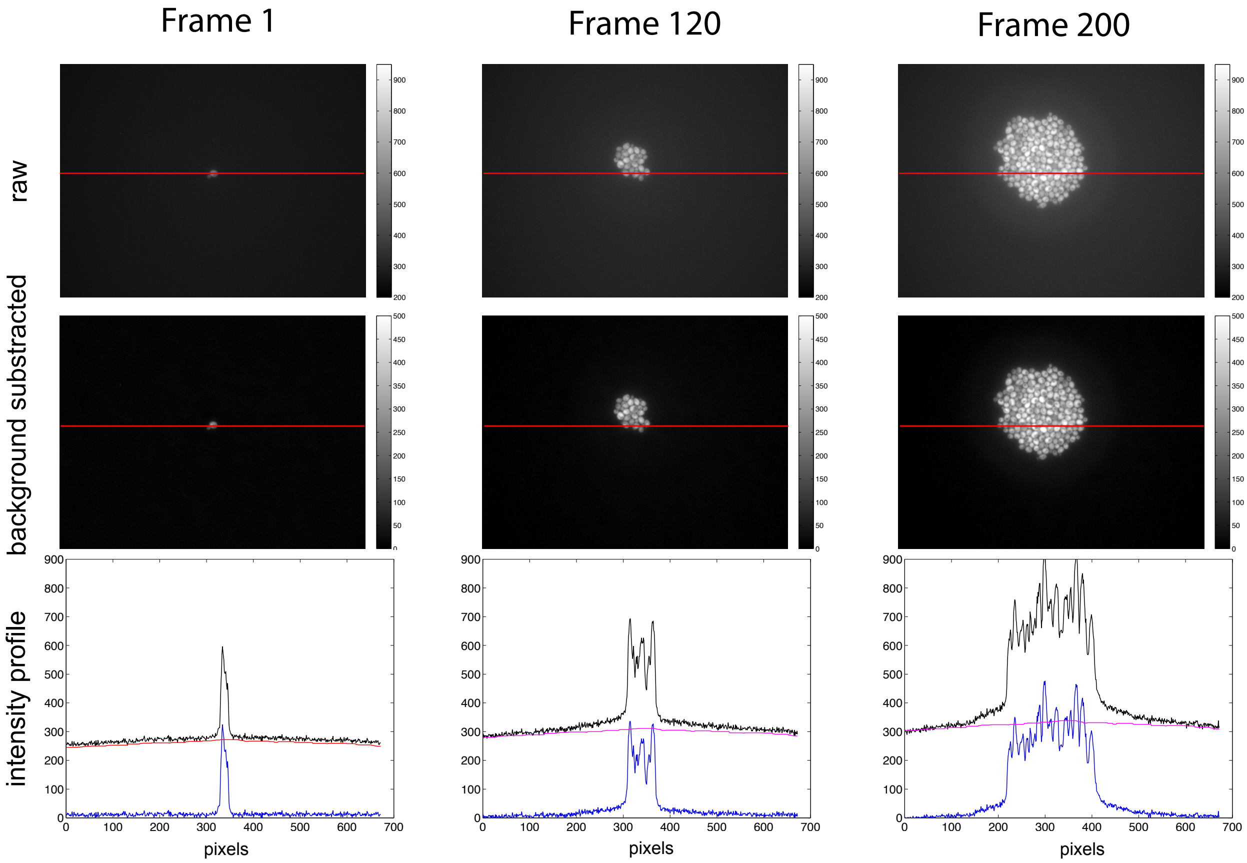

Figure T3: Principle of the background substraction procedure used to remove membrane autofluorescence. Each panel (left, center, right) displays the raw fluorescent image (top), the background substracted image (middle) and some corresponding intensity profiles (bottom) along the displayed red lines. Yeast cells carry a constitutive transcriptional reporter(ACT1-Venus-deg). Black line corresponds to raw image, whereas blue line stands for substracted image. The red and magenta line correspond to the background profile computed from frame 1, scaled to the appropriate level to match the background of the considered frame.

Screenshots

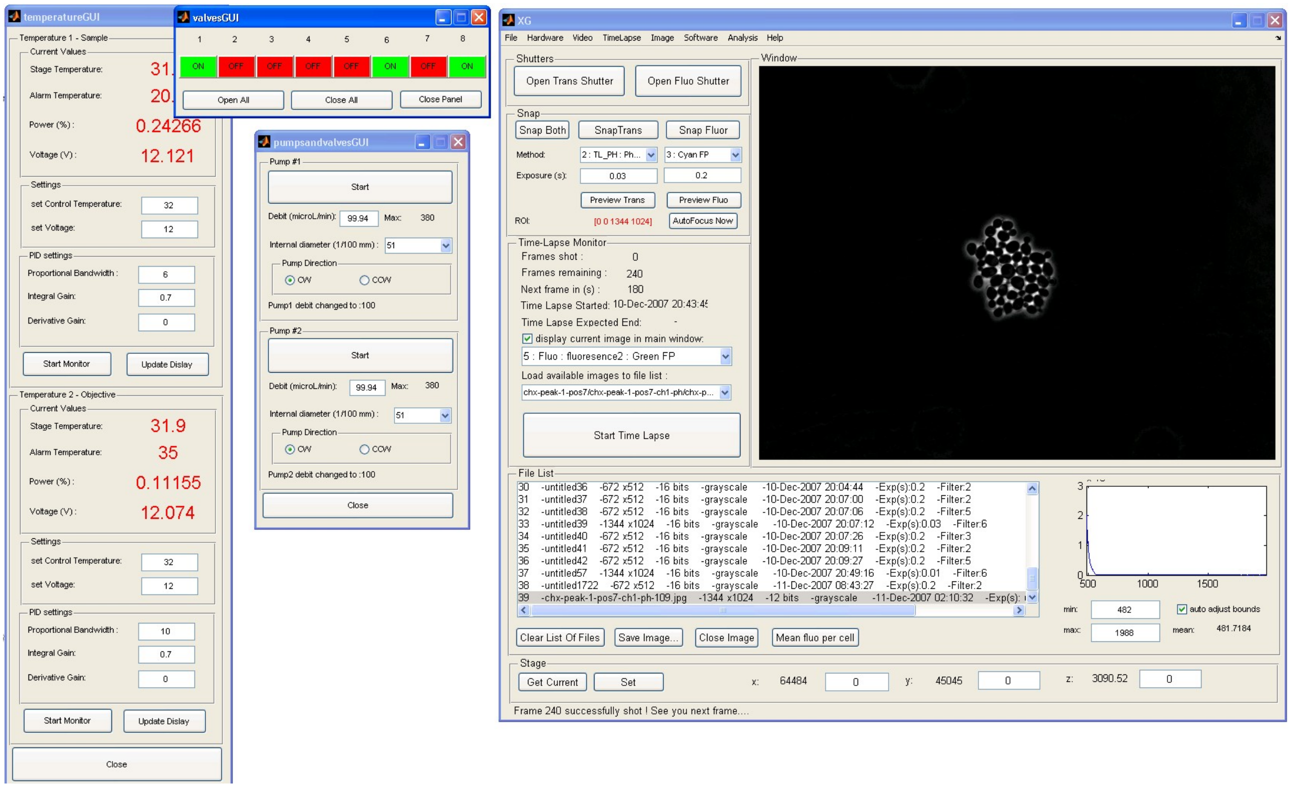

Figure T4: Screenshot of the MATLAB based software to drive hardware, acquire images and control the time-lapse process.

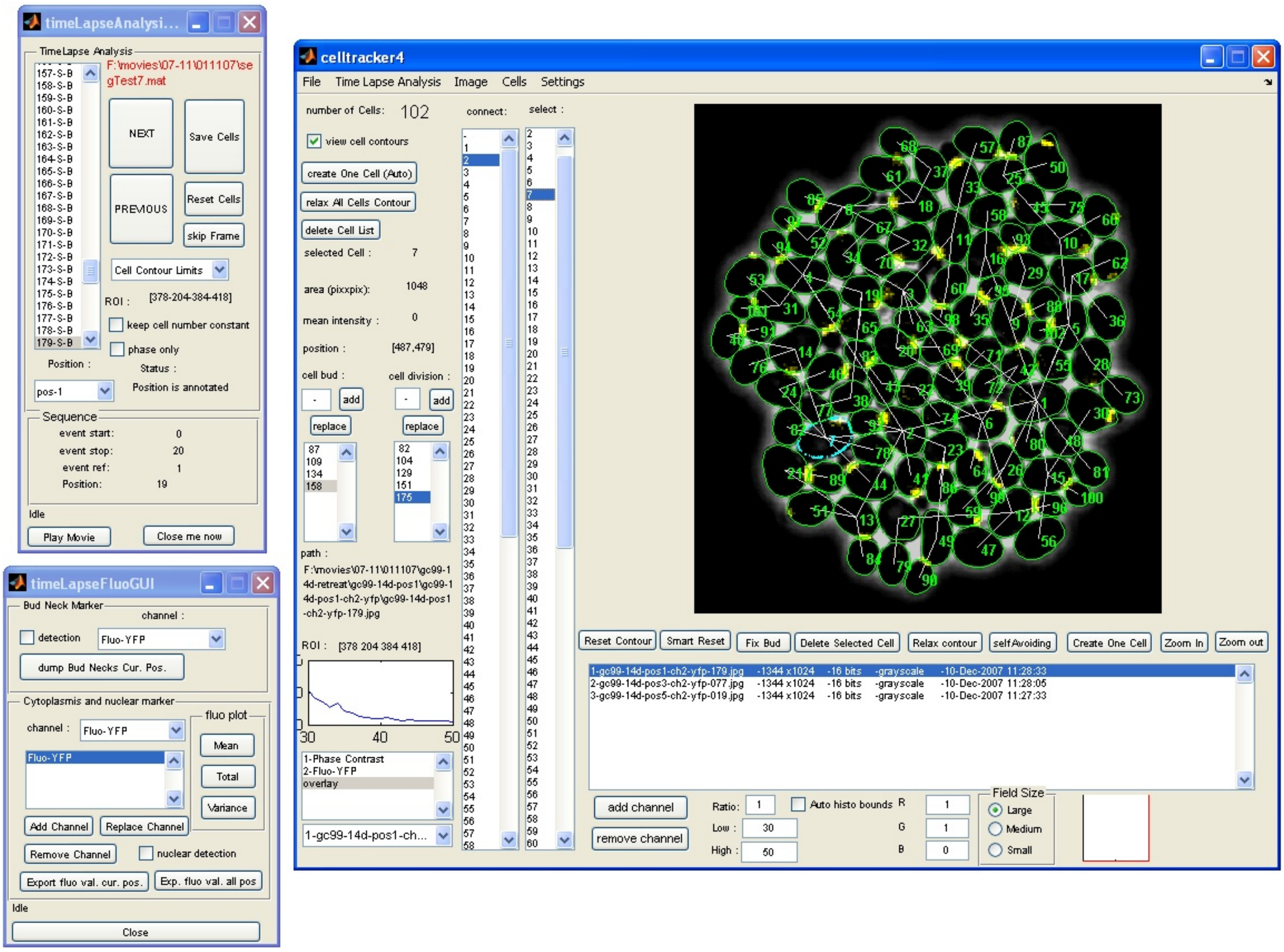

Figure T5: Screenshot of the MATLAB based software to do frame-by-frame annotation of the movie

MODEL FOR THE PULSES OF GENE EXPRESSION

To quantify the dynamics of Venus-deg production from the *MET3* inducible promoter, we used the following minimal model (also used in Ref [8])), as explained in the figure below. Unmatured Venus-deg protein (denoted as P) is produced at an expression rate γ (accounting for both transcription and translation). Then, a maturation step (modeled by a first order reaction with rate β) converts Venus-deg into matured fluorescent protein P*. Last, the protein is degraded at a rate ν, whether it is matured or not.

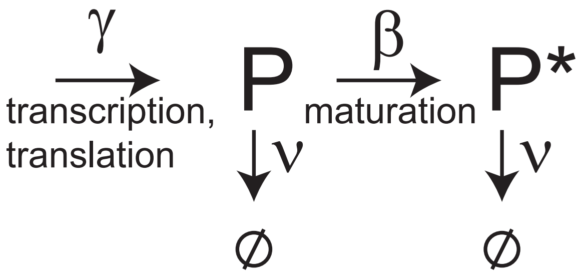

The equation for the dynamics of P and P* are thus :

$$\frac{dP}{dt} = \gamma - (\beta + \nu)P \quad (1)$$

$$\frac{dP^*}{dt} = \beta P - \nu P^* \quad (2)$$

Measurement of Venus maturation time using protein translation block

In order to retrieve the maturation half time $T_\beta = ln(2)/\beta$ indepentently of the other parameters, we monitored fluorescence rise following a translation block for stable Venus under the *MET3* promoter. Since there is no photobleaching evident in Fig. 2c and no dilution since growth has terminated, the fluorescence level should go as :

$$P^*(t) = P_0^* + (P_\infty^* - P_0^*).(1 - e^{-\beta t}) \quad (3)$$

where P_0^* and P_∞^* are the fluorecent protein levels observed when translation is shut-off and at the steady state. A single parameter fit to the cycloheximide block data allowed us to retrieve $T_\beta =$

18.5 ± 0.5 min.

Induction of Venus-deg upon a pulse of inducer

For an inducer pulse of duration τ, equation (1) and (2) gives the evolution of P and P^* as a function of time for $0 < t < \tau$:

$$P(t) = \frac{\gamma}{\beta + \nu} \left(1 - e^{-(\nu+\beta)t}\right) \tag{4}$$

$$P^*(t) = \frac{\beta\gamma}{\beta + \nu} \left[\frac{1}{\nu} \left(1 - e^{-\nu t}\right) + \frac{1}{\beta} \left(e^{-(\nu+\beta)t} - e^{-\nu t}\right)\right]. \tag{5}$$

For $t > \tau$, the fluorescence dynamics should be described by:

$$P^*(t) = \left[P(\tau) + P^*(\tau) - P(\tau)e^{-\beta(t-\tau)}\right] e^{-\nu(t-\tau)}$$

These formulas were fit to the data after removing a lag $\delta \approx 20$ min as explained in the text.

From the fit to the curves corresponding to different cells, we could thus retrieve the cell-to-cell distributions of values of ν and γ, assuming β is a property of the protein and not the cell. It is interesting to note that the steady state fluorescence level for large τ: $P^*_\infty \equiv \frac{\beta\gamma}{\nu(\beta+\nu)}$ is sensitive to the degradation rate ν, quadratically in the extreme case where $\beta << \nu$. This is due to the fact that the fluorescent protein is degraded whether it is matured or not (before and after maturation). Both the degron and the promoter have to be consistently chosen for the expression level desired.

Fluorescence concentration and dilution effect

Since we're not fitting to total protein but fluorescence per area, and since cell area A is growing exponentially with a rate μ, the dynamics of the evolution of unmatured (resp. matured) protein concentration $p \equiv P/A$ (resp $p^* \equiv P^*/A$) is given by :

$$\begin{aligned} \frac{dp}{dt} &= \frac{\gamma}{A} - (\beta + \nu + \mu)p \\ \frac{dp^*}{dt} &= \beta p - (\nu + \mu)p^* \end{aligned} \tag{6}$$

Our growth rate is about half the degradation rate ν, obtained from the fit to the area averaged fluorescence, so not immaterial. However, since most experiments will report the averaged fluorescence

we quoted only $\alpha \equiv \nu + \mu$ in the text. The assumption of balanced exponential growth implies that $\frac{\gamma}{A}$ should be constant.
